# Phospholipase C beta 1 in the dentate gyrus gates fear memory formation through regulation of neuronal excitability

**DOI:** 10.1126/sciadv.adj4433

**Published:** 2024-07-03

**Authors:** Jinsu Lee, Yeonji Jeong, Seahyung Park, Sungsoo Kim, Hyunsik Oh, Ju-Ae Jin, Jong-Woo Sohn, Daesoo Kim, Hee-Sup Shin, Won Do Heo

**Affiliations:** ^1^ Department of Biological Sciences, Korea Advanced Institute of Science and Technology, Daejeon 34141, Republic of Korea.; ^2^ Department of Pathology and Cell Biology, Columbia University, New York, NY 10032, USA.; ^3^ Department of Brain and Cognitive Sciences, Korea Advanced Institute of Science and Technology, Daejeon 34141, Republic of Korea.; ^4^ Center for Cognition and Sociality, Institute for Basic Science (IBS), Daejeon 34141, Korea.; ^5^ KAIST Institute for the BioCentury (KIB), Korea Advanced Institute of Science and Technology (KAIST), Daejeon 34141, Republic of Korea.

## Abstract

Memory processes rely on a molecular signaling system that balances the interplay between positive and negative modulators. Recent research has focused on identifying memory-regulating genes and their mechanisms. Phospholipase C beta 1 (PLCβ1), highly expressed in the hippocampus, reportedly serves as a convergence point for signal transduction through G protein-coupled receptors. However, the detailed role of PLCβ1 in memory function has not been elucidated. Here, we demonstrate that PLCβ1 in the dentate gyrus functions as a memory suppressor. We reveal that mice lacking PLCβ1 in the dentate gyrus exhibit a heightened fear response and impaired memory extinction, and this excessive fear response is repressed by upregulation of PLCβ1 through its overexpression or activation using a newly developed optogenetic system. Last, our results demonstrate that PLCβ1 overexpression partially inhibits exaggerated fear response caused by traumatic experience. Together, PLCβ1 is crucial in regulating contextual fear memory formation and potentially enhancing the resilience to trauma-related conditions.

## Teaser

Spatiotemporal PLCβ1 regulation reveals a gating role in contextual fear memory formation to prevent excessive memory encoding.

## INTRODUCTION

A delicate balance between positive and negative regulators operates at the molecular level to ensure optimal functioning of memory processes under mediation by the hippocampus (*1*–*3*). The intricate coordination of memory processes necessitates the involvement of numerous genes that collaborate to facilitate these cognitive processes. Extensive research on learning and memory has focused on unraveling the mechanisms through which hundreds of genes contribute to memory formation (*4*, *5*). One substantial memory formation mechanism entails the allocation of memory to neurons with relatively increased neuronal excitability (*6*). This hyperexcitable state does not necessarily lead to a positive outcome but, rather, can also have detrimental effects on memory function (*7*, *8*). To maintain normal cognitive functions, the brain limits the consolidation or recall of unwanted memories through suppressive mechanisms that involve elaborate molecular signaling. Increasing the activity of theses mechanisms represses exaggerated learning and memory performance (*4*, *9*, *10*). Consequently, understanding memory modulators is vital for unraveling memory mechanisms and the causes of diseases related to excessive memory retention, such as post-traumatic stress disorder (PTSD) (*11*).

Neurotransmitters modulate complex brain functions by transducing signals between neuronal networks. G protein–coupled receptors (GPCRs) receive the signals and contribute to regulating learning and memory (*12*). Phospholipase C (PLC) is a major downstream enzyme of GPCRs and acts as a critical convergence point for various signal pathways. The PLC acts as an initial enzyme for delivering signals from extracellular information to subcellular organelles by catalyzing the hydrolysis of phosphatidylinositol 4,5-bisphosphate (PIP_2_) and producing the second messengers, inositol trisphosphate (IP_3_) and diacylglycerol (DAG) (*13*). Among the 13 isozymes identified to date, PLC beta (PLCβ) family members receive signal inputs from neurotransmitters that affect memory function and exhibit high-level expression in the brain. Specifically, PLCβ1 was reported to be highly expressed in the cerebral cortex and limbic system (*13*–*15*). Studies on their subcellular expression patterns revealed that PLCβ1 signaling is related to neural function (*13*, *16*). Over the decades, numerous studies have shown that PLCβ1 is relevant to brain functions. For example, its expression is reportedly decreased in psychiatric patients, and homozygous knockout mice reportedly exhibit epileptic behaviors (*17*–*21*). However, the potential contribution of PLCβ1 signaling to hippocampal memory function remains unclear.

Here, we investigated the contextual memory function of PLCβ1 in each hippocampal subregion associated with contextual memory processing in PLCβ1 conditional knockout mice. We report that PLCβ1 knockout in dentate gyrus leads to increased freezing levels during memory retrieval and extinction. To further assess the effects of PLCβ1 depletion, we performed electrophysiological examinations of the intrinsic properties of PLCβ1-deficient neurons and measured the induction of immediate-early genes (IEGs) after fear learning. Given the lack of systems capable of specifically controlling PLCβ1 signaling (*22*), we newly developed an optogenetic system that enables spatiotemporal activation of PLCβ1 signaling. By activating PLCβ1 signaling in dentate gyrus during fear memory acquisition of wild-type mice, we observed the inhibition of IEG induction and a reduction in exaggerated fear response. However, no statistically significant behavioral difference was observed when PLCβ1 signaling was activated during memory recall. Last, in mice exhibiting PTSD-like behavior induced by severe stress, increasing the expression of PLCβ1 repressed the exaggerated fear response. Overall, our results demonstrate that PLCβ1 signaling in neurons of the dentate gyrus controls memory processes by suppressing the encoding of contextual fear memory.

## RESULTS

### PLCβ1 depletion increases fear response and impairs memory extinction

**Fig. 1.**
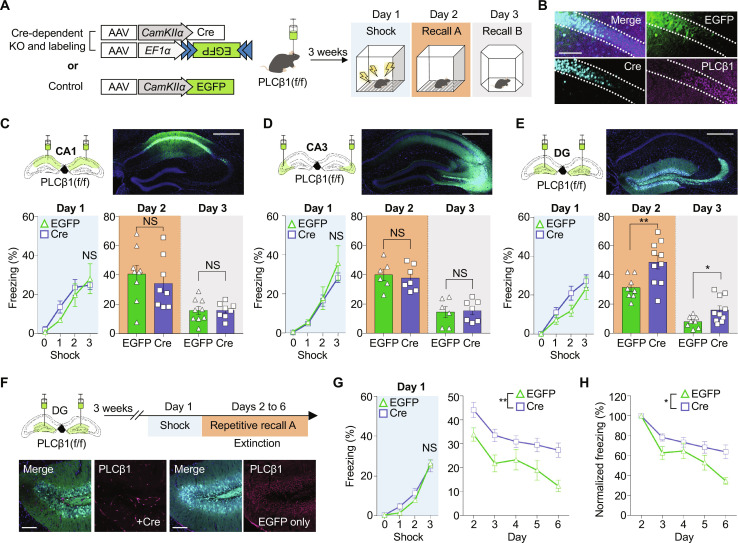
PLCβ1 deficiency in excitatory neurons of the dentate gyrus causes increased fear memory retrieval and impaired extinction. (**A**) Schematic representation of hippocampal subregion-specific PLCβ1 depletion and behavioral paradigm. (**B**) Representative images showing EGFP labeling and PLCβ1 depletion by Cre recombinase in dCA1 pyramidal neurons. Scale bar, 100 μm. (**C** to **E**) Behavioral results of classical fear conditioning after PLCβ1 depletion in dCA1 (C; EGFP, *n* = 9; Cre, *n* = 8), dCA3 (D; EGFP, *n* = 6; Cre, *n* = 7), and dentate gyrus (DG) (E; EGFP, *n* = 8; Cre, *n* = 10). *P < 0.05 and **P < 0.005, unpaired *t* test. Scale bars, 500 μm. (**F**) Schematic of strategy for memory extinction paradigm applied 3 weeks after virus injection, and immunostaining images verifying genetic knockout of PLCβ1 in the DG. Scale bars, 500 μm. (**G** and **H**) Graphs showing the freezing percentage under fear learning (sky) and repetitive exposure to the same context (EGFP, *n* = 11; Cre, *n* = 13). Each freezing level was normalized to that of initial recall (H) [*P < 0.05 and **P < 0.005, two-way repeated measures analysis of variance (ANOVA)]. All data represent means ± SEM. NS, not significant.

To investigate the impact of PLCβ1 on hippocampal contextual memory, we selectively deleted PLCβ1 from the dorsal CA1, CA3, and dentate gyrus regions of PLCβ1-floxed mice using Cre-dependent approaches. For this purpose, we used adeno-associated virus (AAV)–expressing Cre-recombinase driven by the CamKIIα promoter with enhanced green fluorescent protein (EGFP) in the double-floxed inverse open reading frame (DIO) to deplete PLCβ1 and label the neurons (fig. S1). AAVs were bilaterally injected into each hippocampal subregion, and immunostaining was used to verify the colocalization of EGFP and Cre and confirm the Cre-dependent gene knockout. The injected mice were used in classical fear conditioning experiments (Fig. 1, A and B). As a result, while all groups showed normal fear response, only the PLCβ1-deficient mice in the granule neurons of dentate gyrus exhibited an increased freezing during memory recall both in the same context A and even in a novel context B when compared to the control group injected with a virus expressing only EGFP (Fig. 1, C to E). The injection of Cre or EGFP virus into wild-type mice did not affect memory formation or recall (fig. S2).

To assess the persistence of the elevated freezing response during consecutive memory recall, the mice were exposed to the same context for five successive days. This approach was adopted because repetitive exposure to the same context can induce fear memory extinction (*23*). We observed that the increased freezing level was sustained in the Cre-injected group (Fig. 1, F and G). When the freezing level was normalized with respect to the initial level, the rate of fear memory extinction was found to be slower in the PLCβ1-depletion group compared to the control group, indicating that fear memory extinction was impaired in the former group (Fig. 1H). Anxiety level and locomotion of each group did not show any significant between-group difference (fig. S3, A and B). Moreover, to further investigate the contribution of PLCβ1 to contextual fear memory, mice were given tone-paired foot shocks following re-exposure to the conditioning context next day, and then on the following day, they were exposed to a novel context first without the tone and then with the tone (fig. S4A). The results revealed that the PLCβ1-deficient mice showed an increased freezing level, consistent with contextual fear conditioning results (fig S4, B and C). Notably, both groups exhibited an increase in freezing level in response to the tone on day 3, with no significant between-group difference (fig. S4D). These data suggest that mice with PLCβ1 deficiency in the dentate gyrus exhibit an exaggerated fear response and impaired extinction of contextual fear compared to control mice in the same aversive condition.

### PLCβ1 deficiency results in neuronal hyperexcitability

**Fig. 2.**
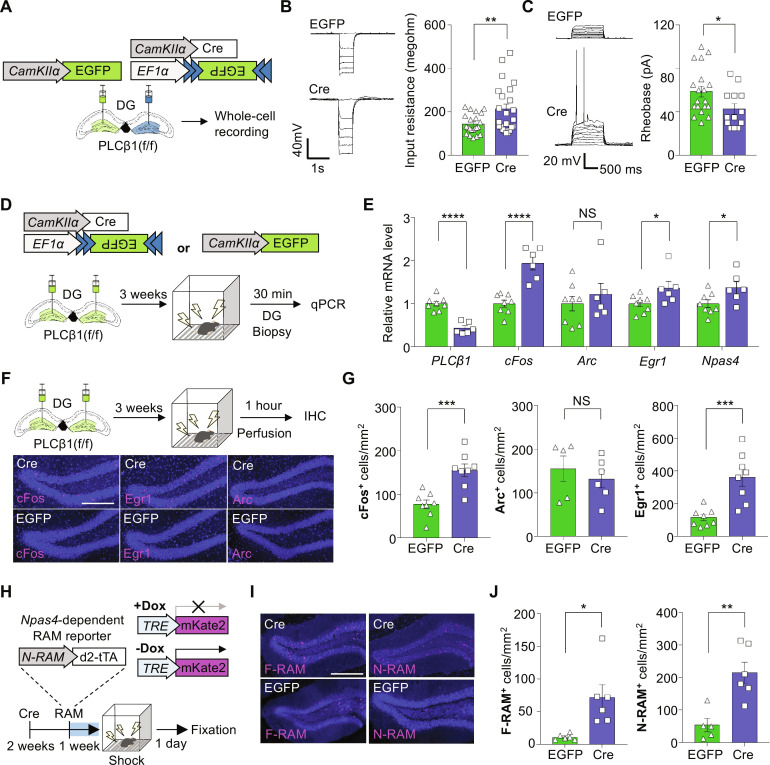
PLCβ1-deleted neurons exhibit hyperexcitability. (**A**) Schematic for using PLCβ1 depletion in one side of the DG to compare intrinsic properties with control values from same mouse brain. (**B** and **C**) Representative subthreshold voltage traces from PLCβ1-depleted or control DG neurons. Higher membrane resistance [B; control (EGFP only), *n* = 22; Cre-expressing neurons, *n* = 23; **P < 0.005, unpaired *t* test] and lower rheobase [C; control (EGFP only), *n* = 19; Cre-expressing neurons, *n* = 13; *P < 0.05, unpaired *t* test] in the PLCβ1-knockout neurons. (**D**) Schematic for quantifying mRNA levels of IEGs after delivery of electric shock. (**E**) Bar graphs showing that mRNA levels are reduced for PLCβ1 and increased for IEGs (EGFP, *n* = 8; Cre, *n* = 6; *P < 0.05 and ****P < 0.0001; unpaired *t* test). (**F**) Schematic for confirming neural activity through immunostaining and representative confocal images of the cFos, Egr1, and Arc. Scale bar, 500 μm. (**G**) Quantification results showing the number of cFos- (EGFP, *n* = 8; Cre, *n* = 8), Arc-positive (EGFP, *n* = 5; Cre, *n* = 6), and Egr1-positive (EGFP, *n* = 8; Cre, *n* = 8) neurons per unit area (mm^2^) after the shock delivery (***P < 0.001, unpaired *t* test). (**H**) Experimental procedure using RAM reporter system. The RAM virus was injected into the same region as the first viral injection, in the presence of doxycycline (blue). The Dox diet was withdrawn 1 day before shock delivery. (**I**) Confocal images of F-RAM^+^ and N-RAM^+^ cells labeled after shock delivery. Scale bar, 500 μm. (**J**) Bar graphs showing populations of F-RAM^+^ and N-RAM^+^ cells per area (mm^2^) in the dentate gyrus after injection of the Cre or EGFP control virus (F-RAM: EGFP, *n* = 6; Cre, *n* = 6; N-RAM: EGFP, *n* = 5; Cre, *n* = 6; *P < 0.05 and **P < 0.005, unpaired *t* test). All data represent means ± SEM.

The dentate gyrus filters excessive excitatory inputs through changes in intrinsic membrane properties (*11*, *24*, *25*). To inspect the cellular mechanisms involved in the behavioral results, we performed whole-cell recording to examine the intrinsic properties of PLCβ1-deficient granule cells. To compare the properties of knockout and control neurons within the same mouse brain, we injected Cre virus into one side of the dentate gyrus and the control EGFP virus into the other side (Fig. 2A). Our results revealed that PLCβ1-knockout cells exhibited a significant increase in membrane resistance and a reduction in rheobase, indicating that they were hyperexcitable state (Fig. 2, B and C, and fig. S5, A to C).

To establish that the exaggerated fear response seen in mice lacking PLCβ1 in the dentate gyrus was due to increased neuronal activity resulting from enhanced excitability, we delivered an electric shock, waited 30 min, and collected the dentate gyrus from the hippocampus (Fig. 2D). We then measured the induction of IEGs, cFos, Egr1, Arc, and NPAS4, which serve as markers for neuronal activity (fig. S6, A and B) (*26*). We found that the mRNA levels of cFos, Egr1, and NPAS4 were increased in PLCβ1-depleted mice compared to control mice (Fig. 2E). Notably, there was no significant difference in the mRNA levels of IEGs in the homecage, supporting our contention that the IEGs were induced a greater degree in PLCβ1-depleted mice after fear conditioning (fig. S7, A and B). To determine whether the population of IEG-positive neurons was increased due to the same foot shock delivery, we used immunohistochemistry to assess the population of IEG-positive neurons in the dentate gyrus at 1-hour after shock (Fig. 2F). We found that PLCβ1-knockout mice in dentate gyrus exhibited more IEG-expressing neurons than control mice (Fig. 2G). To quantify the NPAS4-positive neurons, we injected the dentate gyrus of mice with Cre- or EGFP-encoding virus and, 2 weeks later, with a cFos- or NPAS4-dependent robust activity marking reporter (F-RAM or N-RAM, respectively) (Fig. 2H) (*27*). Under the fear-learning paradigm, we observed more F-RAM^+^ and N-RAM^+^ cells in PLCβ1-knockout mice (Fig. 2, I and J), which is consistent with our mRNA results. Together, our results show that dentate gyrus granule cells lacking PLCβ1 show hyperexcitability, inducing an exaggerated fear response and impaired fear memory extinction.

### PLCβ1 regulates fear memory encoding

**Fig. 3.**
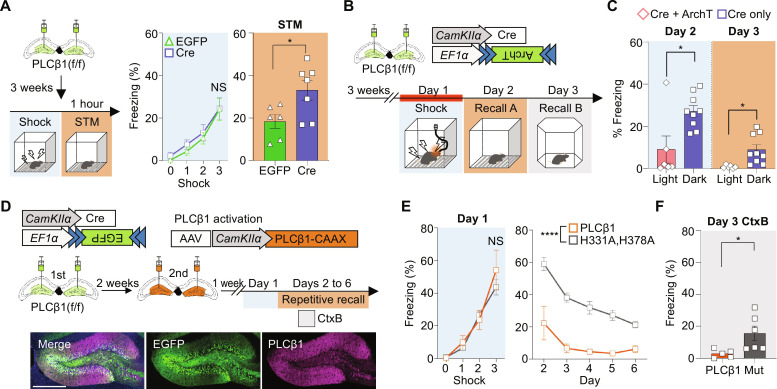
PLCβ1 signaling in the dentate gyrus suppresses neuronal activity and contextual fear memory formation. (**A**) Schematics and graphs showing increased freezing level in short-term memory test (1 hour) performed 3 weeks after injection. (EGFP, *n* = 6; Cre, *n* = 7; *P < 0.05, (left) two-way repeated measures ANOVA and (right) unpaired *t* test). (**B**) Schematic depicting the strategy used for optogenetic inhibition of neuronal activity for fear memory encoding. Mice were subjected to 589-nm laser exposure for a 15-min period including the fear learning period and the fear memory test. (**C**) Behavioral results of classical fear conditioning after inhibition of neural activity (light, *n* = 6; dark, *n* = 9; *P < 0.05, unpaired *t* test). (**D**) Schematic depiction of the fear memory test used to examine the effect of PLCβ1 overexpression after PLCβ1 knockout and immunostaining images showing expression of each construct. (**E** and **F**) Graphs showing fear learning curve (sky) and memory retrieval for repetitive exposures to the same context (E; *P < 0.05, two-way repeated measures ANOVA). (F) Mice were exposed to a novel context at 2 hours after exposure in the fear conditioning chamber on day v3 (F; ****P < 0.000, unpaired *t* test). Scale bar, 500 μm. PLCβ1-KRas4B tail, *n* = 5; PLCβ1(H331A, H378A)-KRas4B tail, *n* = 6. All data represent means ± SEM.

Given our finding that neuronal activity was increased in the dentate gyrus of PLCβ1-depleted mice following fear conditioning, we hypothesized that PLCβ1 plays a role in regulating fear memory encoding. To examine the potential role of PLCβ1 during memory processes, we examined whether the expression of PLCβ1 undergoes changes following contextual fear conditioning and/or recall in the dentate gyrus (fig. S8A). The results revealed that the PLCβ1 mRNA level in the dentate gyrus of wild-type mice was significantly reduced at 1 hour after fear conditioning compared to that in mice kept int their home cage and recovered baseline thereafter (fig. S8B). This suggests that the PLCβ1 is involved in the encoding stage of fear memory. To investigate the impact of PLCβ1 shortly after fear conditioning, we conducted a short-term memory (STM) test 1 hour after fear conditioning of PLCβ1-floxed mice that had received injection of Cre or EGFP virus into the dentate gyrus. Our results revealed that the fear response in the PLCβ1 depletion group was increased compared to that of the control group, providing evidence supporting the involvement of PLCβ1 in contextual memory encoding (Fig. 3A). To further examine whether the increased freezing level in PLCβ1-depleted mice was caused by enhanced neuronal excitability, we inhibited the neuronal activity during fear acquisition by using the light-sensitive opsin, ArchT. We performed optogenetic inhibition of neurons deficient in PLCβ1 during fear conditioning by co-injecting ArchT virus and Cre virus into the dentate gyrus (Fig. 3, B and C). Our results showed that mice in the light group exhibited reduced freezing response during memory retrievals in the fear-conditioning and novel chamber, supporting our hypothesis that increased neuronal excitability by PLCβ1 depletion leads to exaggerated fear memory encoding

### Generation of constitutively active and inactive forms of PLCβ1 enables modulation of PLC activity

To examine whether the enhanced fear response could be reversed by PLCβ1 overexpression, we generated an inactive form as a control. PLC hydrolyzes PIP_2_ through an electrostatic interaction of amino acids within the active site (*28*). Although the crystal structures of other PLC family members, such as PLCδ1 or PLCβ3, have been assessed and their active sites clarified (*28*, *29*), the structure of PLCβ1 is currently lacking. Therefore, we computationally predicted the three-dimensional (3D) structure of PLCβ1 using AlphaFold2 (fig. S9A) and conducted a protein sequence alignment with that of PLCβ3 to identify active sites. Our results confirmed that two histidine residues of PLCβ1, H331 and H378, interact directly with PIP_2_. Accordingly, we used point mutation to replace these two histidine residues with alanine to block the electrical interaction (fig. S9B). We designed a constitutively active form of PLCβ1 by conjugating it with the KRas4B tail (CAAX), which localizes to the plasma membrane and binds the PLCβ1substrate PIP_2_ (*30*). We injected viruses encoding PLCβ1-CAAX or inactive mutant PLCβ1-CAAX into the mouse dentate gyrus, collected tissue samples at 1 week after injection and measured PLC activity accumulated over 2 hours following intraperitoneal injection of LiCl. The results showed that PLCβ1-CAAX induced significantly more PLC activity in the mouse brain compared to EGFP control and the inactive PLCβ1(H331A,H378A)-CAAX (fig. S9C).

### The PLCβ1 knockout-induced alteration of fear memory extinction is rescued by overexpression of active PLCβ1

To assess whether the PLCβ1 knockout-induced delay in fear memory extinction could be reversed by PLCβ1-CAAX overexpression, we injected a Cre-encoding virus into the bilateral dentate gyrus and, 2 weeks later, injected a virus expressing PLCβ1-CAAX or PLCβ1(H331A, H378A-CAAX into the same regions. One week later, the same memory extinction paradigm was conducted to examine the reversal effects of PLCβ1 up-regulation (Fig. 3D). We observed greater freezing level reductions over consecutive context exposures and in a novel context among mice injected with PLCβ1-CAAX compared to the control group, while memory acquisition was normal (Fig. 3, E and F). Together, these behavioral data suggest that mice exhibit an excessive fear response in the absence of PLCβ1 in the dentate gyrus, and re-expression of PLCβ1-CAAX at this location modulated the exaggerated behaviors, supporting the idea that PLCβ1 in the dentate gyrus exerts a suppressive effect on fear memory formation.

### Development of an optogenetic system facilitates specific temporal activation of PLCβ1

**Fig. 4.**
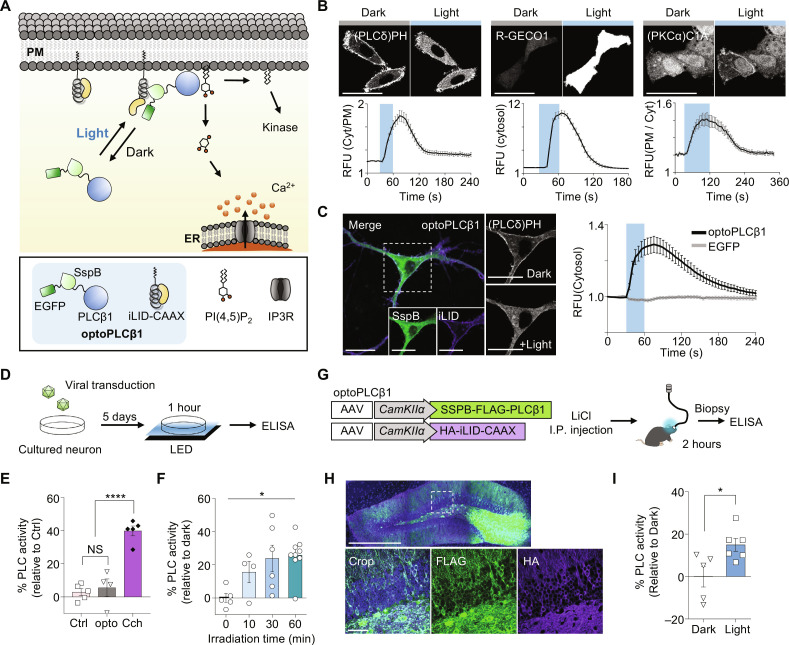
Development and characterization of optogenetic system for specific activation of PLCβ1. (**A**) Schematic design of optoPLCβ1. The optoPLCβ1 translocates from the cytosol to the plasma membrane under blue light illumination and then hydrolyzes PIP_2_ to IP3 and DAG as by-products. (**B**) Representative images and quantitative graphs of optoPLCβ1 in vitro activity under blue light stimulation in HeLa cells [(PLCδ)PH, *n* = 50; R-GECO1, *n* = 47; (PKCα)C1A, *n* = 88]. Blue squares indicate the blue light stimulation time. Scale bars, 50 μm. (**C**) Representative images of hippocampal cultured neuron expressing optoPLCβ1 and time-lapse graph showing translocation of the (PLCδ)PH sensor to the cytosol (optoPLCβ1, *n* = 20; EGFP, *n* = 12). White-dotted region was cropped, and blue squares indicate the blue light stimulation time. Scale bars, 20 μm. (**D**) Schematic of strategy for validating optoPLCβ1 using enzyme-linked immunosorbent assay. (**E**) Bar graph showing basal activity identified through measurement of IP1 production. (control, *n* = 5; optoPLCβ1, *n* = 4; 5 μM carbachol, *n* = 5; ****P < 0.001, one-way ANOVA). (**F**) optoPLCβ1 activity assessed through measuring IP1 production under LED blue light irradiation of various durations (0 min, *n* = 5; 10 min, *n* = 4; 30 min, *n* = 6; 60 min, *n* = 9; *P < 0.05, one-way ANOVA). AAV expressing the HA-iLID-CAAX was used with virus-expressing EGFP-SSPB-PLCβ1 for optoPLCβ1 or with EGFP as controls (E and F). (**G**) Schematic of strategy used to confirm optoPLCβ1 activity in mouse brain. (**H**) Representative immunostaining image showing optoPLCβ1 expression in dentate gyrus. (**I**) Optogenetic activation of PLCβ1 in dentate gyrus (dark, *n* = 5; light, *n* = 6; *P < 0.05, unpaired *t* test). All data represent means ± SEM.

Although we were able to show that exogenous expression of PLCβ1-CAAX accelerated fear memory extinction (Fig. 3, E and F), further exploration of how PLCβ1 functions in memory processes would require the ability to specifically manipulate PLCβ1 signaling. Therefore, we developed an optogenetic PLCβ1 activator by using the improved light-induced dimer (iLID) system to recruit the cytosolic PLCβ1 into the plasma membrane (Fig. 4A). To construct the light-inducible PLCβ1, we first anchored iLID (LOV2 domain with SsrA peptide next to Jα helix) to the intracellular plasma membrane using the CAAX domain of KRas4B. SspB, as a binding partner of iLID, was fused to truncated PLCβ1s in various orientations, and each construct was tested for its ability to activate PLC signaling in HeLa cells. Upon blue light stimulation, only PLCβ1 lacking the C-terminal domain (CTD) showed a response, regardless of the SspB orientation (fig. S10, A and B). This finding is consistent with a previous report that PLCβ1 domains excluding CTD are sufficient for PIP_2_ hydrolysis, whereas the CTD serves as the primary membrane-binding domain responsible for basal activity (*29*). When we observed the subcellular localization of each PLCβ1 construct, full-length PLCβ1 was found in both the plasma membrane and cytosol (fig. S10C). We selected a construct (#2) that showed robust activity, named it as optoPLCβ1, and used it in a two-construct system (Fig. 4A and fig. S10D). To validate the activation of PLCβ1 signaling, we transfected EGFP-SSPB-PLCβ1 and iRFP670-iLID-CAAX into HeLa cells, applied blue light stimulation, detected hydrolysis of PIP_2_ (a substrate of PLCβ1), and increased intracellular Ca^2+^ (modulated through IP_3_ production) and protein kinase C translocation for DAG binding using (PLCδ)PH domain, R-GECO1, and (PKCα)C1A domain, respectively, as indicators (Fig. 4B). We further found that optoPLCβ1 responded to low light density, increased Ca^2+^ at a single-cell level, and could be activated by repetitive blue light stimulation (fig. S11).

Next, we validated the activity of optoPLCβ1 in cultured hippocampal neurons by using the (PLCδ)PH sensor, which underwent translocation of the sensor from the plasma membrane to the cytosol under blue light stimulation (Fig. 4C). To package each optoPLCβ1 construct into AAV, we reduced the vector size by substituting the fluorescent proteins with tag peptides (fig. S12). To confirm that this substitution was appropriate, we measured PLC activity by detecting of IP1 accumulation in cultured hippocampal neurons after viral transfection (Fig. 4D). Notably, there was no significant difference in the basal level of IP1 production between neurons expressing optoPLCβ1 and those expressing EGFP with HA-iLID-CAAX (Fig. 4E). In addition, the amount of IP1 production was significantly elevated as the illumination time increased (Fig. 4F). To examine the activity of optoPLCβ1 in the mouse brain, we injected mixed viruses into the dentate gyrus with implantation of a ferrule. After 3 weeks, the mice were intraperitoneally injected with LiCl, and 2 hours later, photoactivation of optoPLCβ1 was performed (Fig. 4, G and H). We found that the accumulated amount of IP1 was higher in the light group (Fig. 4I). Overall, these results indicate that optoPLCβ1 can specifically induce PLCβ1 signaling both in an in vitro system and in the mouse brain in vivo.

### PLCβ1 signaling inhibits neural activity induction and the formation of contextual fear memory

**Fig. 5.**
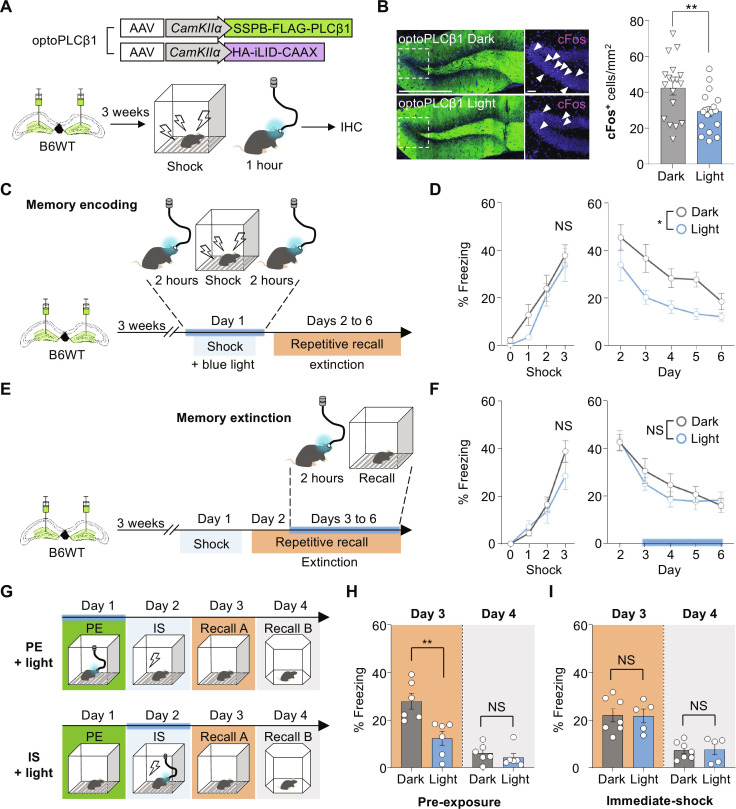
Suppression of cFos induction and fear memory formation by optogenetic PLCβ1 activation. (**A**) Schematic showing strategy for optoPLCβ1 expression and inhibition of neural activity. (**B**) Representative images showing expression of optoPLCβ1 and cFos-positive cells in dentate gyrus. White-dotted region was cropped, and arrows indicate cFos^+^ neurons. Bar graph shows decreased cFos^+^ FLAG^+^ neurons in the light groups (right; dark, *n* = 18; light, *n* = 17; **P < 0.005, unpaired *t* test). Scale bar, 500 μm or 50 μm (for cropped images). (**C**) Schematic of strategy for optoPLCβ1 activation during memory encoding. Light (488-nm laser) was delivered for 2 hours before and after fear learning. (**D**) Graphs showing that there was no significant fear learning and decreased fear memory retrieval upon repetitive exposure in the light group (dark, *n* = 6; light, *n* = 7; *P < 0.005, two-way repeated measures ANOVA). (**E**) Schematics of strategy for optoPLCβ1 activation during memory extinction. (**F**) Graphs showing that there was no significant between-group difference in fear learning or fear memory response (dark, *n* = 9; light, *n* = 7; two-way repeated measures ANOVA). (**G**) Schematic depiction of the strategy used for the context pre-exposure and immediate shock paradigm with optoPLCβ1. (**H** and **I**) Graphs showing that freezing responses were suppressed in the group illuminated with blue light during pre-exposure (PE; dark, *n* = 6; light, *n* = 6), but not significantly altered in that illuminated with blue light during the immediate-shock (IS) period (dark, *n* = 7; light, *n* = 5). (**P < 0.005, unpaired *t* test). All data represent means ± SEM.

To examine whether activation of PLCβ1 signaling in the mouse brain can inhibit neural activity induction during fear memory encoding, we injected AAVs encoding optoPLCβ1 with Cre bilaterally into the dentate gyrus of PLCβ1-floxed mice to deplete endogenous PLCβ1 and induce optoPLCβ1 expression. We then implanted a ferrule to join the optic fiber at one side of the dentate gyrus. Mice were exposed to fear conditioning and 1 hour of optoPLCβ1 activation, allowed to rest in their home cages for 30 min to allow for labeling of cFos^+^ cells, and euthanized (fig. S13A). We found that cFos expression was suppressed in the optoPLCβ1-light group compared to the dark and EGFP-light group. Furthermore, under our experimental conditions, unilateral delivery of blue light was sufficient to activate optoPLCβ1 expressed in both sides of the dentate gyrus (fig. S13, B and C). Consistent with these results, a notable decline in the population of cFos^+^ cells was observed in wild-type mice injected with optoPLCβ1 virus (Fig. 5, A and B).

Next, to demonstrate that PLCβ1 signaling is associated with fear memory suppression in this setting, PLCβ1-floxed and wild-type mice underwent the same surgery as described above and were subjected to the contextual fear conditioning paradigm. To ensure sufficient PLCβ1 signaling activation during fear memory acquisition, mice were exposed to blue light for 2 hours before and after fear memory acquisition (Fig. 5C and fig. S13D). We observed that the light group exhibited a relatively lower freezing level during recall, suggesting that PLCβ1 signaling during memory encoding suppressed the freezing level during memory recall. (Fig. 5D) However, in the PLCβ1-floxed mice, no significant difference in freezing response was observed. This could be attributed to the possibility that the reduction in neuronal activity in highly excitable neurons may not be sufficient to reverse impaired mouse behaviors (fig. S13E). Furthermore, we observed that wild-type mice that received light stimulation during the memory extinction period displayed no significant difference in freezing response (Fig. 5, E and F). However, there was no significant reduction in the initial memory recall. Considering that there was no significant difference in the freezing responses in PLCβ1-floxed mice (fig. S13E), it remains possible that optoPLCβ1 did not sufficiently induce PLCβ1 signaling in mouse brains in vivo, especially in this behavioral paradigm, whereas PLCβ1 signaling was robustly activated within our in vitro experiments (Fig. 4 and fig. S11).

To further assess the contribution of PLCβ1 signaling to suppressing contextual memory formation, we conducted the context pre-exposure and immediate shock paradigm with temporal activation of PLCβ1 expression using optoPLCβ1. Exploration enables mice to form a contextual representation that is essential for their ability to associate the context with the shock. Without this encoding, no association is established upon immediate shock exposure and the fear response fails to form. However, pre-exposure facilitates the context-shock association (*31*). Thus, we used optoPLCβ1 to selectively induce PLCβ1 signaling during pre-exposure (PE group) for assessment of context encoding or during immediate shock (IS group) for assessment of the ability to associate shock with context (Fig. 5G). The PE groups exhibited reduced freezing levels, indicating that PLCβ1 signaling suppresses context encoding (Fig. 5H), whereas there was no significant change in the IS groups, suggesting that PLCβ1 signaling does not affect memory recall once the initial contextual representation is formed (Fig. 5I). This finding is consistent with our observation that wild-type mice exposed to light stimulation during the memory extinction period displayed no significant difference in freezing responses (Fig. 5F). Overall, these results suggest that PLCβ1 signaling-related suppressive mechanisms affect the memory-encoding process to prevent the consolidation of fear memory.

### PLCβ1 expression modulates resilience to exaggerated fear response by stress-induced fear learning

**Fig. 6.**
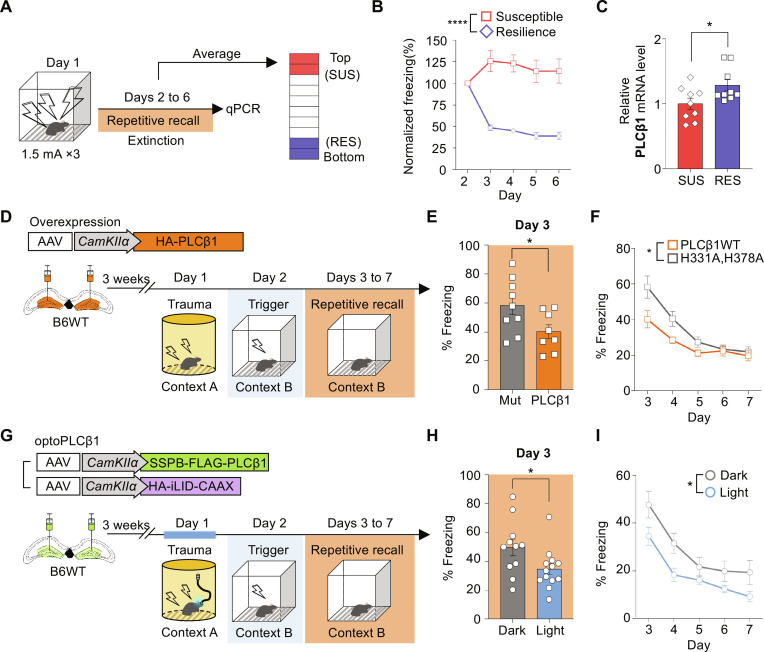
PLCβ1 expression modulates resilience to traumatic experience. (**A**) Schematic of memory extinction paradigm with strong foot shock. Mice were arranged on the basis of their average normalized freezing level and categorized into the susceptible (top 25%) and resilient (bottom 25%) groups. (**B**) Normalized freezing levels of each group (SUS, *n* = 9; RES, *n* = 9; ****P < 0.0001, two-way repeated measures ANOVA). (**C**) PLCβ1 mRNA level is higher in the resilient group (SUS, *n* = 9; RES, *n* = 9; *P < 0.05, unpaired *t* test). (**D**) Schematic of strategy for induction of traumatic experience and amelioration of PTSD-like behavior by PLCβ1 overexpression. (**E** and **F**) Graphs showing alleviation of fear responses in the PLCβ1-overexpression group on day 3 (E) and during repetitive recall (F) [PLCβ1(H331A,H378A), *n* = 9; PLCβ1, *n* = 8; *P < 0.05, two-way repeated measures ANOVA). (**G**) Schematic depicting the strategy used for optogenetic activation of PLCβ1 during a traumatic experience (**H** and **I**) Graphs showing a reduction in freezing level on the first recall (H) and amelioration of PTSD-like behavior (I) [dark, *n* = 11; light, *n* = 13; *P < 0.05, (H) unpaired *t* test and (I) two-way repeated measures ANOVA]. All data represent means ± SEM. qPCR, quantitative PCR.

Depletion of PLCβ1 resulted in an exaggerated fear response, generalized fear memory (Fig. 1E), and impaired fear memory extinction (Fig. 1, G and H), which are core symptoms commonly observed with neuronal hyperexcitability (Figs. 2 and 3) in animal models of PTSD and with neuronal hyperactivity in individuals with PTSD (*32*–*35*). Moreover, several studies have shown a connection between PLCβ1 and brain disorders such as epilepsy and psychiatric disorders involving the cortex (*13*, *16*). Given that hyperactivity of the dentate gyrus has been associated with stress susceptibility and PTSD (*36*, *37*), we hypothesized that PLCβ1 is associated with suppressive mechanisms that can support resilience to PTSD-like behavior following traumatic experiences. To test this hypothesis, we first conducted contextual fear conditioning with intense electric shocks to induce PTSD-like behavior, observed fear memory extinction retention (*38*, *39*), and classified the mice into susceptible and resilient groups based on the average normalized fear level during memory retrieval, excluding the first recall. The top 25% of mice with the highest normalized freezing level were classified as susceptible, while the bottom 25% with the lowest fear level were classified as resilient using a previously reported scheme (Fig. 6A) (*40*). We compared the PLCβ1 mRNA level between the two groups (Fig. 6B). The susceptible group had a relatively lower mRNA level of PLCβ1, indicating that PLCβ1 expression may contribute to resilience to PTSD-like behavior (Fig. 6C).

We next hypothesized that the suppressive mechanism of PLCβ1 would filters out excessive contextual memory encoding to prevent an exaggerated fear response that could lead to PTSD-like behavior (*7*). To assess the potential involvement of the suppressive role of PLCβ1 in a behavioral paradigm with severe shock conditions and examine a potential association with PTSD, we conducted a stress-enhanced fear learning paradigm in which mice were exposed to a traumatic stressor in specific context A and subsequently to a mild stressor as a trigger of the traumatic memory in a different context (context B). We tested the paradigm after injecting mice with PLCβ1 in the dentate gyrus (Fig. 6D). One day after triggering the traumatic experience in context B, the fear response triggered by the shock was suppressed in the PLCβ1-overexpressed group (Fig. 6, E and F). However, the reduction was not sustained during five consecutive days of memory recall. To further validate the behavioral findings and examine whether PLCβ1 signaling is involved in traumatic memory encoding, we injected optoPLCβ1 into the dentate gyrus and performed the same paradigm with blue light exposure during traumatic experience (Fig. 6G). We observed that the light group exhibited reduced freezing level compared to the dark group. This is consistent with the results obtained for PLCβ1 overexpression and demonstrates that the development of PTSD-like behavior is at least partly alleviated by PLCβ1 up-regulation (Fig. 6, H and I).

## DISCUSSION

Neuronal communication for cognitive function involves diverse GPCR signals that commonly activate PLCβ1, which thus acts as a convergence point for extracellular signal inputs to trigger intracellular signaling cascades (*12*). PLCβ1 is mainly expressed in pyramidal neurons of the cerebral cortex and hippocampus (*14*). Previous studies found that PLCβ1 null-knockout mice exhibited epilepsy, hyperlocomotion, startle response, abnormal social ability, and deficits in fear (*19*, *41*). However, the reported results were mostly related to cortical function and were obtained using homozygous PLCβ1-knockout mice, making it difficult to interpret the precise function of PLCβ1 in the hippocampus. Here, we used a conditional knockout mouse (Cre-dependent PLCβ1 depletion) to investigate the functions of PLCβ1 in each hippocampal subregion. Intriguingly, only mice depleted of PLCβ1 in the dentate gyrus exhibited relevant effects, including increased freezing level during retrieval and a slower memory extinction rate. Although depletion of PLCβ1 from the dentate gyrus was found to have opposing effects on learning of fear memory and extinction memory, the high level of fear retrieval in PLCβ1-deficient mice before extinction might influence their impaired fear extinction (*42*). Previous experiences significantly influence our learning process, either hindering further learning, as observed in instances of proactive interference, or facilitating it by providing a framework for rapid incorporation (*43*). The enhancement of one specific type of memory might reallocate cognitive resources or neural circuitry and thereby affect the acquisition or consolidation of other memories. The heightened contextual fear memory might compete for cognitive resources or attention, potentially interrupting or interfering with other memory learning types, including motor learning and spatial memory (*8*, *44*). The present and previous results thus demonstrate that chronically enhanced neuronal excitability does not necessarily lead to enhanced learning.

Our findings of increased freezing levels 1 hour after fear conditioning in PLCβ1-depleted mice (Fig. 3A) and reduced freezing responses in optoPLCβ1-light groups exposed to blue light during memory acquisition (Figs. 5D and 6, H and I) support the idea that PLCβ1 signaling plays a more substantial role in the initial encoding or early stages of memory formation in the dentate gyrus. We speculate that the lack of significant behavioral alterations among mice deficient for PLCβ1 in CA1 or CA3 may reflect the following: (i) Although the hippocampal subareas are closely related in both physical and functional aspects, they exhibit distinct reactions to external influences. First, although the hippocampal subareas physically and functionally closely related, they exhibit distinct reactions to external influences (*45*–*49*). Notably, PLCβ1 signaling induces region-specific effects on memory processes, as evidenced by reports that agonist responses vary across different brain regions and null knockout of PLCβ1 in mice leads to decreased muscarinic M1 receptors in the cortex and muscarinic M1/M4 receptors in the hippocampus (*19*, *50*). Given this, we speculate that local depletion of PLCβ1 in CA1 or CA3 may not directly affect contextual fear memory processing. Meanwhile, PLCβ1 in the dentate gyrus likely regulates memory consolidation processes that occur after contextual information signals are received from the entorhinal cortex. (ii) Given that null knockout affects intracellular signaling or voltage-dependent cationic conductance in CA3 regions (*44*, *51*), we cannot exclude the possibility that compensatory mechanisms present in CA1 and CA3 mitigate the effects of PLCβ1 knockout but are less effective or absent in the dentate gyrus, potentially leading to differences in the behavioral phenotypes of knockout mice (*52*). A comprehensive understanding of the molecular, cellular, and circuit-level mechanisms in each hippocampal region will be essential for deciphering the specific impact of PLCβ1 knockout on contextual fear memory.

This is in line with previous reports suggesting that PLCβ1 signaling is associated with muscarinic acetylcholine receptors (mAChRs) in the hippocampus, and mice null for the M1 subtype of mAChR showed enhanced contextual fear memory (*19*, *53*). However, the behavioral phenotypes reported previously were not identical to those observed in mice PLCβ1-deficient mice, indicating the involvement of other receptors associated with PLCβ1 signaling in fear memory function. While further investigation is necessary to confirm this, it is evident that PLCβ1 in the dentate gyrus regulates contextual fear memory through suppressive mechanisms.

To address potential cellular mechanisms underlying the effects seen in the present study, we recorded intrinsic properties of PLCβ1-deficient granule neurons. They exhibited a significant rise in membrane resistance and a decrease in the rheobase, indicating that they had entered a high-excitability state (*25*). To further examine whether the neural activity of the highly excited neurons could be more robustly evoked by fear conditioning, we measured the induction of IEGs (cFos, Egr1, Arc, and NPAS4; used as representative neuronal activity markers) at the mRNA and protein levels. We found that PLCβ1 depletion increased activation of a subset population of depleted neurons activated by fear conditioning rather than the entire population of depleted neurons. This may be influenced by pre-existing inhibitory mechanisms that manifest in sparse active population of neurons, since the dentate gyrus is fundamentally characterized as a silent region for memory processing (*25*, *54*). Our results suggest that the exaggerated fear response seen in mice with PLCβ1 depletion in the dentate gyrus reflects an increase in the population of hyperexcitable neurons, leading to excessive neuronal activation.

PLC interacts with various signaling receptors, including cholinergic, noradrenergic, and metabotropic glutamatergic receptors, which are essential for N-methyl-D-aspartate receptor (NMDAR)–dependent long-term depression (LTD). This process is crucial for spatial memory encoding in the dentate gyrus and in the CA1 region, linking LTD and long-term potentiation (LTP) with spatial memory processing (*55*). Decreased NMDAR-LTD is associated with hyperexcitability, as evidenced by significantly increased input resistance and reduced rheobases for firing action potentials in the thalamus and primary sensory neurons (*56*, *57*). However, NMDAR activation is essential for LTP and LTD, which are both critical for learning and memory but exhibit opposing effects (*55*, *58*, *59*). NMDAR signaling triggers the influx of calcium into postsynaptic neurons, which distinct calcium signaling patterns differing for each process (*60*). Notably, some receptors coupled to the PLC pathway are essential for the induction of NMDAR-LTD (*61*, *62*). Thus, the altered excitability seen in our results could be influenced by interactions between NMDAR and other receptors that couple directly with PLCβ1, such as mGluR5 (*63*, *64*). In addition, memory learning induces synaptic and nonsynaptic changes, including heightened intrinsic excitability, which can reshape neuronal encoding of subsequent information (*43*, *65*). For example, NMDARs at synapses of both excitatory and inhibitory neurons may affect inhibitory neuronal function and GABAergic synapse plasticity on excitatory neurons (*66*). Moreover, PLCβ1 activation can regulate the production of DAG, which is required for endocannabinoid release and has been implicated in LTD induction. Disruption of this pathway, as seen in PLCβ1 knockout mice, may lead to excessive neural activity and related conditions, such as epileptic hyperexcitability, by disrupting the balance between excitatory and inhibitory signaling in the brain (*19*, *62*, *67*, *68*).

Accumulated studies have reported that the dentate gyrus receives contextual information from the entorhinal cortex and filters out unwanted memory for memory consolidation through changes in excitability that regulate membrane conductance and enable this structure to function as a “gatekeeper” of contextual memory (*2*, *25*, *69*). However, there is a lack of studies examining how the dentate gyrus regulates memory formation at the molecular level. A previous report found that the enzyme activity and protein level of PLCβ1 were reduced in response to fear conditioning (*70*)_._ Here, we report that the PLCβ1 mRNA level in the dentate gyrus of wild-type mice was significantly decreased at 1 hour after fear conditioning but returned to baseline thereafter (fig. S8B). This may suggest that contextual information inputs reduce PLCβ1 expression and activity to facilitate fear memory encoding, and there is a subsequent up-regulation of PLCβ1 to prevent storage of excessive memory.

To support our hypothesis that PLCβ1 contributes to the memory-encoding step, we needed to undertake experiments involving the temporal manipulation of PLCβ1 signaling. Although there are existing systems capable of controlling the GPCR/Gq/PLC pathway and thereby modulating pathways upstream of PLCβ1, these systems are not specific to PLCβ1 because the activation of GPCR signaling could conceivably alter other signal pathways (*22*, *71*). Thus, we here set out to develop an optogenetic activator of PLCβ1. Toward this end, we generated several truncated forms of PLCβ1 by removing each domain and used activity assessments to identify an optimal construct. Of the truncated constructs, only CTD-deleted PLCβ1(1-816) induced PLC signaling. This is consistent with previous results suggesting that the PH, EF, catalytic X-Y, and C2 domains of PLC enzymes are necessary for PIP_2_ hydrolysis as a catalytic core (*29*, *72*). These findings were obtained using chemical agonists, but these chemicals might also interact with Gαq and have unexpected effects and would therefore interrupt understanding the function of each domains in PLCβ1. However, our optogenetic system was designed to induce PLCβ1 variants to undergo plasma membrane translocation and bind to PIP_2_ as a substrate, without involving any exogenous factor. The CTD helps anchor PLCβs to the plasma membrane and has Gαq-interacting domains (*73*). Consistent with this, we observed that EGFP-tagged full-length PLCβ1 was localized in the plasma membrane and cytosol, whereas constructs lacking the CTD were expressed only in the cytosol (fig. S5C). Therefore, deletion of the CTD might reduce basal activity by dissociating optoPLCβ1 from the plasma membrane and removing the Gαq interaction site.

Retrieval of contextual fear memory formation was increased in PLCβ1-deficient mice, whereas retrieval of contextual fear memory extinction was unaltered, implying that PLCβ1 may not merely serve as memory suppressor but rather may play a specific role in memory processes. Using optoPLCβ1, we were able to specifically stimulate PLCβ1 signaling during fear memory learning or memory retrieval. This enabled us to observe that there was a significant reduction of the fear response when PLCβ1 was activated during memory encoding, but there was no significant difference when PLCβ1 was activated during the repetitive context exposure. These results support our hypothesis that PLCβ1 signaling in the dentate gyrus modulates the learning and encoding of contextual fear memory (Fig. 5, D and E). Although we anticipated that mice exposed to blue light during memory acquisition would show significantly decreased fear response beginning with the first memory recall, the statistical analysis of multiple comparison revealed that a significant reduction was not observed until day 3. Considering that there was no significant alteration in the freezing responses of PLCβ1-floxed mice (fig. S13E), we cannot exclude the possibility that optoPLCβ1 did not induce sufficient PLCβ1 signaling in mouse brains in vivo, especially in this behavioral paradigm, whereas PLCβ1 signaling was robustly activated during our in vitro experiments (Fig. 4 and fig. S11). We note, however, that mice injected with a constitutively active form of PLCβ1 exhibited a notable induction of PLC activity and reduced freezing levels during memory recall, but no alteration of the freezing level during memory acquisition (Fig. 3, D and E). These results, together with those obtained with constitutively activated PLCβ1 (PLCβ1-CAAX), suggest that induction of PLCβ1 signaling causes more notable effects than overexpression of PLCβ1.

In a contextual fear-conditioning paradigm, mice initially form a representation of the context by exploring the context before it becomes associated with the unconditioned stimulus (foot shock). These processes are termed context encoding and context conditioning, respectively. Context encoding precedes context conditioning and is essential for the ability of the mouse to form an association between the stimulus and the context (*74*). However, mice that have been pre-exposed to the conditioning chamber are able to associate the two stimuli and exhibit a freezing response. Coupling the pre-exposure and immediate-shock paradigm with the precise temporal activation of optoPLCβ1 during a specific memory process enabled us to separate the effects of PLCβ1 on the encoding of context versus its association with shock. Overall, the findings support our hypothesis that PLCβ1 signaling in the dentate gyrus acts as a memory suppressor during memory encoding after fear acquisition.

Several studies have linked PLCβ1 with brain disorders, such as epileptic seizure, depression, and schizophrenia in the cortex (*13*, *16*, *21*). Abnormal PLCβ1 expression has been observed in the post-mortem brains of psychiatric patients and individuals who committed suicide (*17*, *18*). Recent evidence suggests that the hippocampal dentate gyrus is associated with stress susceptibility and PTSD. The latter is a mental disorder that has a high prevalence rate in the global population and characteristic symptoms of fear generalization and impaired memory extinction with neuronal hyperactivity (*34*, *36*, *37*, *75*). These symptoms are consistent with those observed in mice with PLCβ1 depletion in the dentate gyrus. Given that IEG expression is related to memory function, excessive activation of underlying processes may cause maladaptive fear and lead to psychiatric diseases, such as PTSD. While most individuals will experience one or more traumatic episodes in their lifetime, only a small fraction will develop PTSD symptoms due to individual differences in stress susceptibility. Although there have been efforts to identify memory-regulating genes associated with psychiatric diseases, studies are still needed to identify relevant genes with significant effects related to PTSD (*76*, *77*). Here, we used a fear memory extinction task and found that the mRNA level of PLCβ1 was lower in the PTSD-susceptible mouse group and that up-regulation of PLCβ1 decreased the freezing level in the PTSD mouse model generated by traumatic experience. This suggests that PLCβ1 up-regulation might be associated with enhanced resilience to PTSD. Going forward, further studies on the association of PLCβ1 with PTSD might provide notable insights into the susceptibility to this psychiatric disease.

## MATERIALS AND METHODS

### Animals

All mice were housed under a 12-hour light/12-hour dark cycle at room temperature and 40% humidity. PLCβ1 flox/flox mice used in this study was obtained from Hee-Sup Shin group in Institute for Basic Science (Korea) and were maintained in a C57BL/6J background and used for surgery at 8 weeks of age. Mice with the same genetic background were used as wild-type mice. Mice are randomly assigned to each injection group and behavioral test. For primary hippocampal neuronal culture, embryonic day 18 embryos of either sex were obtained from pregnant Sprague-Dawley female rats (14 weeks old; used immediately after purchase from Koatech). Animal experiments were conducted according to the guidelines of the Institutional Animal Care and Use Committee of Korea Advanced Institute of Science and Technology (KAIST).

### Cell lines

HeLa and human embryonic kidney (HEK) 293 T (American Type Culture Collection) cells were maintained in Dulbecco’s modified Eagle’s medium (DMEM; catalog no. 11965092, Gibco) supplemented with 10% fetal bovine serum (FBS; Gibco) at 37°C with 10% CO_2_. These cell lines were confirmed to be contamination-free using a BioMycoX Mycoplasma PCR detection kit (catalog no. D-50, CellSafe).

### Plasmid construction

To generate the optoPLCβ1 construct, the iLID-CAAX(KRas4B) sequence was polymerase chain reaction (PCR)–amplified from the Venus-iLID-CAAX (Addgene, #60411) plasmid, digested with Eco RI and Bam HI, and inserted into pEGFP-C1 (Clontech). The EGFP was replaced with iRFP682 by Nhe I and BsrG I digestion and ligation to generate plasmid CMV∷iRFP682-iLID-KRas4B tail. The EGFP-SspB-PLCβ1 plasmid was generated by inserting SspB (Addgene, #60415; a binding partner of iLID and human PLCβ1) into pEGFP-C1 vector using Age I/Bsr GI and Bsp EI/Bam HI digestion and ligation. Plasmids encoding the PLCβ1 variants were generated by replacing full-length PLCβ1 with truncated PLCβ1 sequences that had been PCR-amplified and subjected to restriction and ligation with Bsp EI and Bam HI. The same sequences of truncated PLCβ1 were also inserted to pEGFP-C1, and SspB was amplified and inserted into the N or C termini of EGFP-PLCβ1 plasmids to generate SspB-EGFP-PLCβ1 and EGFP-PLCβ1-SspB, respectively.

To reduce the insert size of optoPLCβ1 for AAV packaging, the fluorescent proteins in each vector, iRFP682 and EGFP, were replaced with the tag peptides, hemagglutinin (HA) and FLAG, respectively, using Gibson assembly Cloning (NEB). Then, HA-iLID-CAAX and SSPB-FLAG-PLCβ1 were cloned into pAAV-CamKIIα-hChR2(H143R)-mCherry (Addgene, #26975). Considering the limited size capacity of AAV, the short form of WHV posttranscriptional regulatory element (WPRE) was obtained from pAAV-CW3SL-EGFP (Addgene, #61463) and used to replace the original WPRE sequence of pAAV-CamKIIα-hChR2(H143R)-mCherry. To label F-RAM^+^ or N-RAM^+^ cells, the viral plasmids of pAAV-F-RAM-d2tTA-TRE-mKate2 and pAAV-N-RAM-d2tTA-TRE-mKate2 were purchased from Addgene (#140274 and #140275, respectively). The constitutively active PLCβ1 was generated by performing PCR-based addition of Kras4B-tail sequences (Addgene, #60411) after the Y816 of PLCβ1 and inserting the generated sequence into pAAV-CamKIIα-HA-iLID-CAAX. To perform point mutation of H331 and H378 of PLCβ1 to alanine, PLCβ1 was PCR-amplified with primers including the desired H331A or H378A mutations, and the mutated PLCβ1 was used to replace the wild-type version in HA-PLCβ1-CAAX via the Bsp EI and Bam HI sites to generate pAAV-CamKIIα-HA-PLCβ1(1-816)-CAAX and pAAV-CamKIIα-HA-PLCβ1(1-816)(H331A,H378A)-CAAX.

### Cell culture and transfection

For live-cell imaging, HeLa cells (8 × 10^3^ per well) were plated to a 96-well plate (#89626, iBidi) and transfected with LTX lipofection (Invitrogen) for 1 day. Briefly, 200 ng of plasmids was mixed with PLUS and LTX reagents at a 1:1:2 ratio in 50 μl of optiMEM (catalog no. 31985-070, Thermo Fisher Scientific) and loaded to each well.

For primary hippocampal neuronal culture, we dissected hippocampi from embryonic brains in Hank’s balanced salt solution (HBSS; catalog no. 14185-052, Gibco) with 10 mM Hepes (catalog no. 15630-080, Gibco). Trypsinized hippocampi were washed with FBS serially diluted in the HBSS solution. Dissociated neurons were filtered through a 70-μm cell strainer (catalog no. 352350, BD Falcon), and 3.5 × 10^4^ cells per well were immediately plated to a 24-well plate (catalog no. P24-1.5H-N, Cellvis) containing NM10 plating medium (10% horse serum (catalog no. 16050-122, Gibco), 2% GlutaMAX (catalog no. 35050-061, Gibco), and 1% antibiotic antimycotic solution (catalog no. SV30079.01, Cytiva Hyclone) in neurobasal medium). The plate was incubated for 1 hour at 37°C in a humidified 5% CO_2_ atmosphere, and the NM10 medium was exchanged to neurobasal medium containing B27 Supplement (catalog no. 17504-044, Gibco) (maintaining medium). The maintaining medium was replaced with 40% fresh medium every 3 days. All media and sera were purchased from Gibco unless otherwise indicated. Cultured neurons were used at 11 to 16 Days In Vitro (DIV) for live-cell imaging. For transfection, 600 ng of plasmids was mixed with PLUS and LTX reagent (catalog no. 15338-100, Invitrogen) at a 1:1:1 ratio in 50 μl of neurobasal medium. Half of the neuronal medium was removed from each well (and retained), and the above-described liposome mixture was added dropwise to each well. After 1 hour, the medium was fully replaced with that retained in the prior step plus 50% fresh medium. All cells were imaged at 16 to 24 hours after transfection.

### Production of AAV

AAV was generated in our laboratory using a protocol that was previously described in detail (*78*), with some modification. In brief, HEK293T cells were transfected with plasmids including the gene of interest, a packaging plasmid (AAVDJ/8), and a helper plasmid at a ratio of 1:4:2. Polyethylenimine (PEI) in Dulbecco’s PBS (DPBS) was mixed with the plasmids at a ratio of 2.5:1, and this mixture was applied to cells through dropwise pipetting. The culture medium (10% FBS in DMEM) was changed after 4 hours of transfection. At 3 days after transfection, the culture media were collected and replaced with fresh media. At 5 days after transfection, the culture media and cells were harvested and centrifuged for polyethylene glycol (PEG) precipitation. The cell pellets and precipitated PEG were resuspended in SAN digestion buffer containing SAN High Quality enzyme (catalog no. 70920-202, ArcticZymes Technology), loaded on an iodixanol gradient, and ultracentrifuged at 350,000*g* for 1 hour. The 40% iodixanol layer (containing the AAV) was removed from the tube using a 5-ml syringe, washed with DPBS, concentrated to 150 to 250 μl using an Amicon tube (100,000 MWCO; Millipore), and stored at −80°C.

### Animal stereotactic surgery

Surgery was performed on 8-week-old mice. Mice were anesthetized with Avertin (200 mg/kg; 2,2,2-tribromoethonol in PBS), and AAVs were injected into each hippocampal subregion (stereotaxic atlas: CA1, anteroposterior (AP), −2.0/mediolateral (ML), ±1.4/dorsoventral (DV), 1.25; CA3, AP, −2.3/ML, ±2.6/DV, 1.9; dentate gyrus, AP, −2.0/ML, ±1.3/DV, 1.7). For all injections, 0.2 μl was applied over 3 min. For PLCβ1 knockout in transgenic mice, AAVDJ/8-CamKIIα∷Cre, AAVDJ/8-EF1α∷DIO-EGFP, and AAVDJ/8-CamKII∷EGFP were diluted to 1 × 10^12^ GC/ml in PBS. For optogenetic inhibition, AAV2/9-CAG-FLEX-ArchT-GFP (1x10^13^ GC/ml) was injected with Cre virus. To deliver optoPLCβ1 viruses, AAVDJ/8-CamKIIα∷HA-iLID-CAAX (1 × 10^12^ GC/ml) and AAVDJ/8CamKIIα∷SSPB-FLAG-PLCβ1 (1 × 10^13^ GC/ml) were injected into each brain region. For labeling of neuronal activity using the RAM system, AAVDJ/8-F(or N)-RAM∷d2tTA-TRE-mKate2 viruses were diluted to 1 × 10^12^ GC/ml and injected at 2 weeks after the initial injection. One day before the second injection, mice were given a Dox diet of 1 g/kg; after the second injection, this was changed to a Dox diet of 40 mg/kg, which was supplied until 24 hours before the initiation of contextual fear conditioning.

For photoactivation, immediately after virus injection, a 200-μm-diameter zirconia cannula optic ferrule (Doric) was implanted in one side of the dentate gyrus (AP, −2.0/ML, +1.3/DV, 1.1) and fixed with dental cement. After surgery, mice were housed in cages wherein perforated opaque dividers separated the mice while allowing them to communicate with one another. After experiments, genotyping was performed using a Dyne direct PCR kit (#BN430, Dyne Bio) with primer pairs for flanking the loxP site of exon 11: 5′-TGGCCTTTTCCCAAAGACTCAGGTCAG-3′ and 5′-TCACGAAAGCATTCTAATTGTGCCCTG-3′.

### Measurement of PLC activity

Primary hippocampal neurons (6 × 10^4^ cells per well) were plated to a 24-well plate. For expression of optoPLCβ1, AAV-CMV∷HA-iLID-CAAX(KRas4B) and AAV-CMV∷EGFP-SSPB-PLCβ1 were mixed at 50,000 multiplicity of infection (MOI) in 50 μl of DPBS. Half of the neuronal medium was removed and retained, and the above-described mixture was applied with fresh medium. After 1 day of transduction, the medium was fully replaced with the retained medium plus enough fresh medium to recover the volume. PLC activity was measured by assessing IP1 accumulation using an IP-one enzyme-linked immunosorbent assay (ELISA) kit (catalog no. 72IP1PEA, Cisbio) according to the manufacturer’s instructions. In brief, the neuronal media was fully changed to stimulation buffer, and optoPLCβ1 was activated by blue light using a 470-nm light-emitting diode (LED; Live Cell Instrument) as follows: 300 μW/mm^2^, 10 s of irradiation every 60 s for 1 hour. For chemical activation of PLC signaling, Carbachol was diluted to 5 μM in the provided stimulation buffer. After PLC stimulation, the sample was lysed, transferred to a microplate, and reacted with 3,3′5,5′-tetramethylbenzidine for 13 min. Optical density (OD) was measured at 450 nm with optional correction at 610 nm, using a VersaMax microplate reader (Molecular Devices).

For measuring PLC activity in mouse brains, each mouse was given an intraperitoneal injection of 10 μM LiCl/400 μl saline, anesthetized with Avertin (200 mg/kg), and exposed to blue light stimulation (5 mW/mm^2^, 20 mHz, 20% duty cycle) for 2 hours. The dentate gyrus was collected with a 1-mm biopsy punch (catalog no. BPP-10F, Kai Medical) in a dark room, and fixed in liquid nitrogen. The AAV injection site was checked for the coexpressed EGFP (as a marker) using a dual fluorescent protein flashlight (catalog no. DFP-1, NightSea). Samples without the EGFP signal were excluded. The samples were stored at −80°C until ELISA experiments. To ensure the use of equal amounts of brain samples, each thawed sample was lysed in 80 μl of PRO-PREP (catalog no. 17081, iNtRON), supplemented with 50 μM LiCl, the protein concentration was measured, and equal amounts were used. The subsequent steps were conducted according to the manufacturer’s instructions (Cisbio).

### Behavioral tests

#### Contextual fear conditioning and extinction

All mice were handled in the behavior room for 5 min/day for 3 days before experiments and habituated in the room for at least 10 min before the behavior test. For activity-dependent labeling experiments and fear conditioning, mice were habituated in context A (described below) for 3 min, exposed to three electric foot shocks (0.75 mA, 2 s), and promptly returned to their home cage at 1 min after the last foot shock. For the memory-recall test, mice subjected to fear learning were returned to context A on the following day (day 2), and the freezing responses were recorded over 5 min (day 2). On day 3, either the mice were exposed to a novel place (context B, described below) and fear generalization was tested by monitoring freezing for 5 min, or the mice were returned to the same context (context A), and fear extinction was tested by monitoring freezing during 5 min for five consecutive days. Freezing levels were analyzed using FreezeFrame3 (ActiMetrics). Context A was a white transparent acryl box (18.5 cm by 18.15 cm by 32 cm) with an electrical floor grid. Context B was a hexagonal metal chamber (30 cm in width). For traumatic fear conditioning, mice received a 1.5-mA shock.

For photoactivation of optoPLCβ1, a 473-nm laser was used at 5 mW/mm^2^ and 20 mHz with a 20% duty cycle (10s on/ 40s off) for 2 hours before-and-after fear learning or for 2 hours before context exposure for the successive memory recalls on Days 3 to 6 in the same context. For optogenetic neuronal inhibition during memory acquisition, mice were exposed to a sustained 589-nm laser 10 mW/mm^2^ 5 min before, during, and after fear acquisition. After fear learning, mice were exposed to the fear-conditioning context on day 2 and the novel context on Day 3.

#### Stress-enhanced fear learning

Enhanced fear learning through traumatic experience was conducted based on a protocol that was previously described in detail (*79*), with modification. Mice were single-caged 3 days before experiencing trauma. For traumatic experience, each mouse was put into a yellow cylinder of 20 cm in diameter and subjected to 10 electric shocks (1 mA, 1 s) that were given randomly over 1 hour. Mice of a no-trauma group were put into the same context but not given any shock. One day later, mice were placed in a white transparent acryl box (18.5 cm by 18.15 cm by 32 cm) with 40% diluted mouthwash for 3 min, received one trigger shock (1 mA, 1 s), and promptly returned to their home cage after 30 sec. The memory-extinction paradigm was conducted by exposing the mice to the same white transparent box for 5 min per day for 5 days. Freezing level was recorded.

#### Pre-exposure and immediate shock paradigm

Mice were handled in the behavioral chamber for 5 min/day for 3 days and then pre-exposed to fear-conditioning context A and allowed to explore for 10 min on day 1. On the following day, the mice were given immediate shock delivery, wherein they were returned to the same context, exposed for 8 s, and then given a foot shock (0.75 mA, 2 s) (*31*). On day 3, the mice were exposed to the fear-conditioning chamber for memory recall during 5 min. On day 4, they were exposed to novel context B. Temporal photoactivation was achieved using a 473-nm laser at 5 mW/mm^2^ and 20 mHz with a 20% duty cycle (10-s on/40-s off) for 5 min during pre-exposure, including 2 hours before and after the pre-exposure (PE group), or 2 hours before and after the immediate shock (IS group).

#### Auditory fear conditioning

Tone-paired fear conditioning was performed as described in our previous study (*80*). In summary, mice were habituated for 2 min and then exposed to a tone (30s, 60 dB) paired with a foot shock (2 sec, 0.75 mA) three times at 2-min intervals. The mice were returned to their home cages 90 sec after the last conditioning section. On Day 2, the mice were reintroduced to the conditioning chamber A, and on Day 3, they were exposed to a novel context B on Day 3 without a 3 min tone, followed by a 3 min tone.

#### Open-field test

The open-field test was performed in a square acryl chamber (40 cm by 40 cm by 40 cm). Each mouse was placed at the center of the chamber and allowed to move freely for 30 min. The total distance moved and the time spent in the central area (20 cm by 20 cm) were analyzed using EthoVision XT (Noldus).

### Electrophysiology

For whole-cell recording of PLCβ1-depleted neurons, PLCβ1-floxed mice were injected with AAVDJ/8-CamKIIα∷Cre and AAVDJ/8-EF1α∷DIO-EGFP on one side of the dentate gyrus and AAVDJ/8-CamKIIα∷EGFP on the other side. The patch clamper was blind to the viral condition of either side of the dentate gyrus. Mice were anesthetized with isoflurane and transcardially perfused with a cutting solution (220 mM sucrose, 26 mM NaHCO3, 2.5 mM KCl, 1 mM NaH2PO4, 5 mM MgCl2, 1 mM CaCl2, and 10 mM glucose; pH 7.3 to 7.35). After decapitation, the whole brain was promptly immersed in ice-cold cutting solution saturated with carbogen. Horizontal sections of 300 μm in thickness were obtained using a vibratome (VT1200s, Leica) and then incubated in a storage solution (123 mM NaCl, 26 mM NaHCO3, 2.8 mM KCl, 1.25 mM NaH2PO4, 1.2 mM, MgSO4, 2.5 mM CaCl2, and 10 mM glucose; pH 7.3 to 7.35) at 34°C before recording. Recording was performed using a recording solution (126 mM NaCl, 26 mM NaHCO_3_, 2.8 mM KCl, 1.25 mM NaH_2_PO_4_, 1.2 mM MgSO_4_, 2.5 mM CaCl_2_, and 5 mM glucose; pH 7.3 to 7.35). Glass pipettes had resistances of 3 to 6 megohm and were filled with an internal solution (120 mM potassium gluconate, 10 mM KCl, 10 mM Hepes, 5 mM EGTA, 1 mM CaCl_2_, 1 mM MgCl_2_, and 2 mM Mg–adenosine triphosphate; pH 7.29). Recordings were discarded when an access resistance was above 35 megohm either before or after recordings were completed. Input resistance was measured by −40-pA increments of hyperpolarizing currents of 500-ms duration, from 0 to −200 pA.

### Immunohistochemistry

Cardiac perfusion was performed with cold DPBS, and brain samples were obtained and fixed in 4% paraformaldehyde overnight at 4°C. Each fixed brain was cut into 50-μm coronal slices using a VT1200s vibratome (Leica). Two or three slices were distributed to a 24-well plate (catalog no. 30024, SPL) containing blocking buffer of 300 μl per well (5% normal goat serum diluted in 0.3% Triton X-100 PBS) and incubated for 1 hour at room temperature (RT). The buffer was replaced with blocking buffer containing the appropriate dilution of primary antibody and incubated at 4°C overnight or for 2 days. The slices were rinsed five times with 0.3% PBS-X, secondary antibodies diluted in blocking solution were applied to each well, and incubation was continued for 2 hours at RT. The slices were washed five times with 0.3% PBS-X and mounted onto slide glasses using VECTASHEILD with 4′,6-diamidino-2-phenylindole (DAPI) (catalog no. H-1200, Vector Laboratories). The following primary antibodies were used at the indicated dilutions: anti-PLCβ1 (1:500; Santa Cruz Biotechnology, sc-5291), anti-DYKDDDK tag (1:500; BioLegend, 637301), anti-HA tag [1:1000; Cell Signaling Technology (CST), 2367S], anti-Cre recombinase (1:1000; Abcam, ab190177), anti-cFos (1:1500; CST, #2250), anti-Egr1 (1:1000; CST, #4154), and anti-Arc (1:1500; SYSY, 15600). The secondary antibodies were as follows: anti-Rat immunoglobulin G (IgG) conjugated with Alexa Fluor 488 (1:1000; Invitrogen, A-11006), anti-Rabbit IgG conjugated with Alexa Fluor 594 (1:1000; Invitrogen, A-11012), anti-Rabbit IgG conjugated with Alexa Fluor 647 (1:1000; Invitrogen, A-21244), and anti-Mouse IgG conjugated with Alexa Fluor 594 (1:1000; Invitrogen, A-11005).

### Confocal imaging and analysis

For live-cell and immunostaining imaging, we used a Nikon A1R confocal microscope (Nikon Instruments) equipped with a CO_2_ and temperature incubation system (Live Cell Instruments). The fluorescence intensity changes of mCherry-(PLCδ)PH, R-GECO1, and (PKCα)C1A-FusionRed over time were analyzed using the “Time Measurement” tool of the software provided with the microscope or otherwise clarify. For whole photoactivation of the optogenetic system, cells were coimaged using 488-nm and 561-nm laser to detect expression and stimulate the optoPLCβ1. To analyze the IEG-positive cells of the mouse dentate gyrus, at least four hemispheres were used for quantification after “denoise” processing. The neuronal activity marker–positive cells that were also DAPI^+^ EGFP^+^(or FLAG^+^) were counted manually by an observer who was blind to each group. Mice with off-target expression following injection into the dentate gyrus were excluded from the analysis.

### Western blot and quantitative PCR

For measuring protein or mRNA levels in brain tissues, mice were anesthetized with Avertin and euthanized, and mouse brains were collected and cut into about 1-mm coronal slices using a stainless-steel brain matrix (Zivic Instruments). Virus expression was confirmed using a flashlight (NightSea). Each slice was frozen onto a blade using dry ice, the dentate gyrus was collected with a 1-mm biopsy punch, and the collected punches were immediately frozen in liquid nitrogen.

To obtain proteins, dentate gyrus was lysed using PRO-PREP (50 μl per tube; iNtRON) and a homogenizer and then centrifuged at 15,000*g* (4°C) for 5 min to remove tissue debris. For SDS–polyacrylamide gel electrophoresis (SDS-PAGE), equal amounts of protein (30 μg) were mixed with 5X SDS-PAGE loading buffer (catalog no. S2002, BIOSESANG), boiled at 95°C for 5 min, and resolved on a 10-well Bolt 4 to 12% Bis-Tris Plus Gel (catalog no. NW04120BOX, Invitrogen). The resolved proteins were transferred to an iBlot nitrocellulose membrane (catalog no. IB301001, Invitrogen), which was incubated with Tris-buffered saline (TBS)–blocking buffer (catalog no. 927-60001, LI-COR) for 1 hour on a shaker and then with the primary antibodies, such as anti-PLCβ1 (1:500; Santa Cruz Biotechnology, sc-5291) and anti-GAPDH (1:5000; Invitrogen, MA5-15738), for 2 days at 4°C. The membrane was washed with 0.1% Tween 20 in TBS (TBS-T) buffer, incubated with IRDye-conjugated secondary antibodies diluted in blocking buffer, and rinsed with TBS-T buffer. The following secondary antibodies were used: anti-Rabbit IgG with 680RD (1:20,000; LI-COR, 926-68071) and anti-Mouse IgG with 800CW (1:20000; LI-COR, 926-32210). Fluorescence-labeled protein bands were detected using an Odyssey CLx apparatus (LI-COR).

RNA was purified using an RNA mini kit (catalog no. 12183018A, Ambion) according to the manufacturer’s instructions. During experiments, all samples were maintained on ice. Equal amounts of purified RNA (150 ng) were applied to synthesize cDNA using a PrimeScript RT reagent kit with gDNA Eraser (catalog no. RR047A, Takara). For quantitative PCR, the samples were amplified and measured using the SYBR Green Realtime PCR Master Mix (catalog no. QPK-201, TOYOBO) and a CFX Opus 96 Real-time PCR system (BIO-RAD). The used primer sequences are listed in table S3.

### Quantification and statistical analysis

All data are analyzed blindly and presented as the means ± SEM. We used the term “significant” only when there was statistically significant difference or when there was no statistically significant difference. For statistical comparisons, we used two-sided unpaired *t* test or one-way or two-way analysis of variance (ANOVA) with repeated measure. For post hoc analysis, we used simultaneous multiple comparisons. The statistical scores and details are presented in tables S2 and S3. Statistical analysis was performed with GraphPad Prism 7. Statistical significance was determined as follows; *P < 0.05, **P < 0.01, ***P < 0.001, ****P < 0.0001, and NS = not significant (*P* > 0.05).
