# Supplementary material for: Phospholipase C beta 1 in the dentate gyrus gates fear memory formation through regulation of neuronal excitability
Source: Sci Adv. 2024 Jul 3;10(27):eadj4433. doi: 10.1126/sciadv.adj4433 (PMC11221510; doi:10.1126/sciadv.adj4433)
Supplement: Supplementary file 1 — Figs. S1 to S13 Tables S1 to S3 [file sciadv.adj4433_sm.pdf]

Science Advances
AAAS

Supplementary Materials for

Phospholipase C beta 1 in the dentate gyrus gates fear memory formation through regulation of neuronal excitability

Jinsu Lee *et al.*

Corresponding author: Won Do Heo, wondo@kaist.ac.kr

Sci. Adv. **10**, eadj4433 (2024)
DOI: 10.1126/sciadv.adj4433

This PDF file includes:

Figs. S1 to S13
Tables S1 to S3

Fig. S1

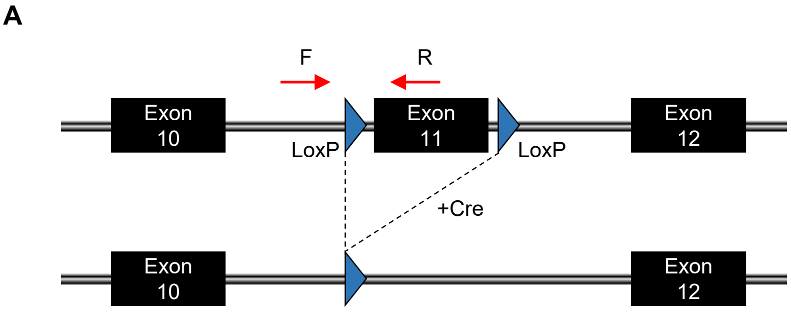

***Primers for genotyping of PLCβ1*-floxed mouse**	
Forward	TGGCCTTTTCCCAAAGACTCAGGTCAG
Reverse	TCACGAAAGCATTCTAATTGTGCCCTG

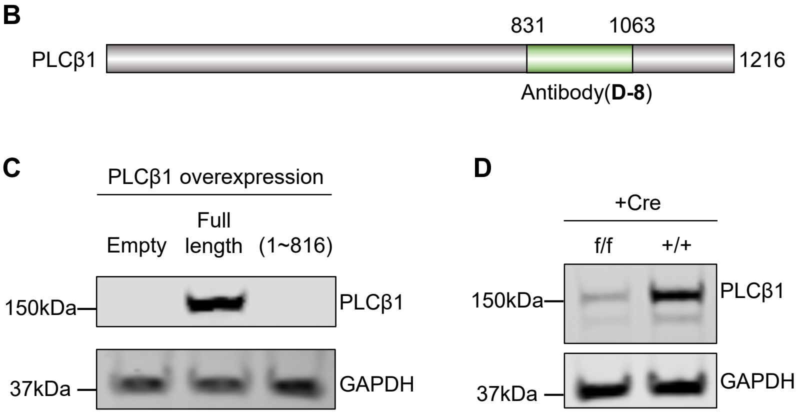

Fig. S1. Details of PLCβ1-floxed mouse and Cre recombinase-dependent gene knockout
(A) Graphical diagram of PLCβ1-floxed construct for Cre-dependent depletion in mice, and primer sequences used for screening. (B) Graphical diagram of monoclonal PLCβ1 antibody(D-8). Green box represents the recognition site of the antibody. (C) Antibody specificity testing of HeLa cells transfected with vectors encoding full-length PLCβ1 or antibody binding region-deleted PLCβ1, or without transfection (empty). (D) Immunoblotting result showing Cre-dependent PLCβ1 knockout in mouse brain. AAVDJ/8-CamKIIα::Cre was bilaterally injected into hippocampal CA1 region of a PLCβ1-floxed mouse (f/f) and a wild-type mouse (+/+).

Fig. S2

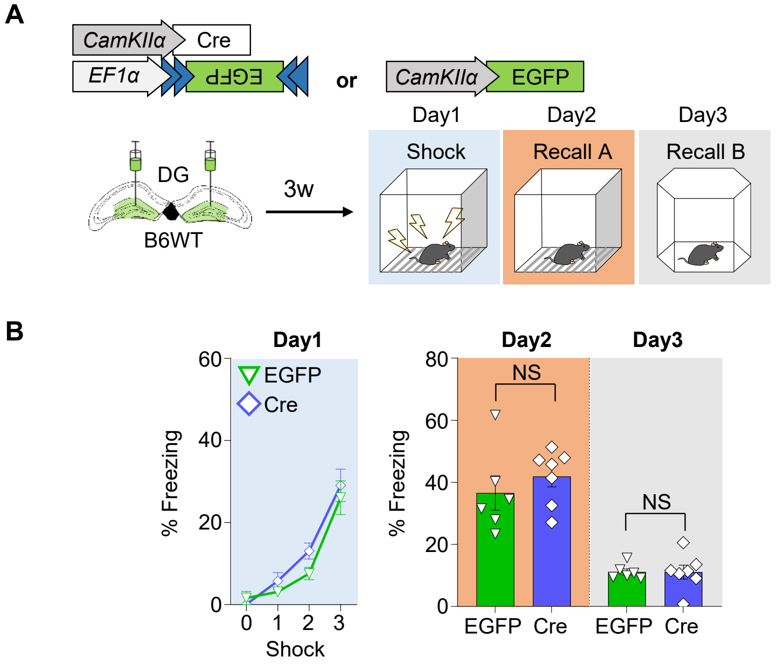

Fig. S2. Effects of Cre overexpression on freezing levels in wild-type mice
(A, B) Schematic (A) and graphs (B) of contextual fear conditioning results. The freezing level of memory retrieval on Day2 and Day3 showed no significant difference between the Cre-injected and control groups. (EGFP n=6, Cre n=7; NS, not significant, unpaired t-test). All data represent means ± s.e.m.

Fig. S3

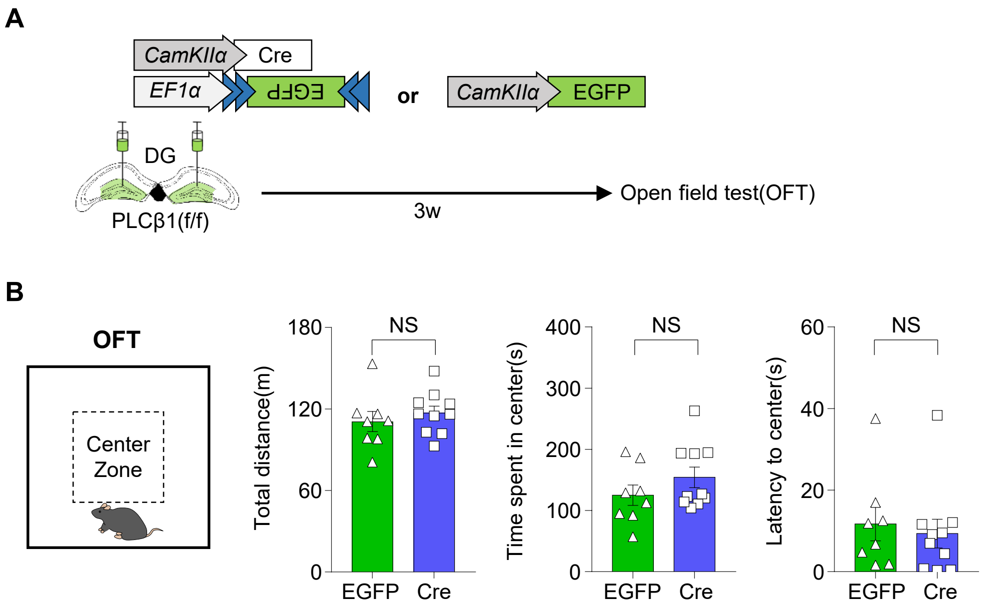

Fig. S3. Analysis of locomotion and anxiety in mice depleted of PLCβ1 in the dentate gyrus
(A-B) Schematic and bar graphs of open-field test results. Cre-injected group and control group showed no significant difference in the total distance, time spent in the center, and latency to center (EGFP n=8, Cre n=10; NS, not significant, unpaired t-test). All data represent means ± s.e.m.

Fig. S4

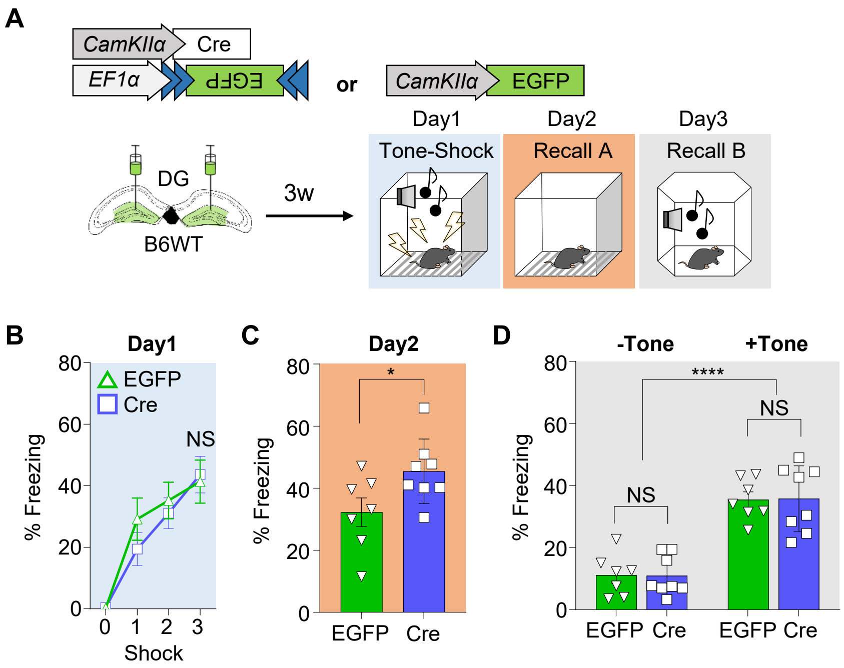

Fig. S4. Auditory fear conditioning test in mice depleted of PLCβ1 in the dentate gyrus
(A-D) Schematic depiction and bar graphs of auditory fear conditioning results. The freezing percentages under fear learning (B) and recall in context A (C; *$p<0.05$, unpaired t-test) on Day 2 and in novel context B for 3 min without a tone followed by 3 min with a tone (D). (EGFP n=7, Cre n=8; ****$p<0.0001$; NS, not significant, one-way ANOVA with multiple comparisons). All data represent means ± s.e.m.

Fig. S5

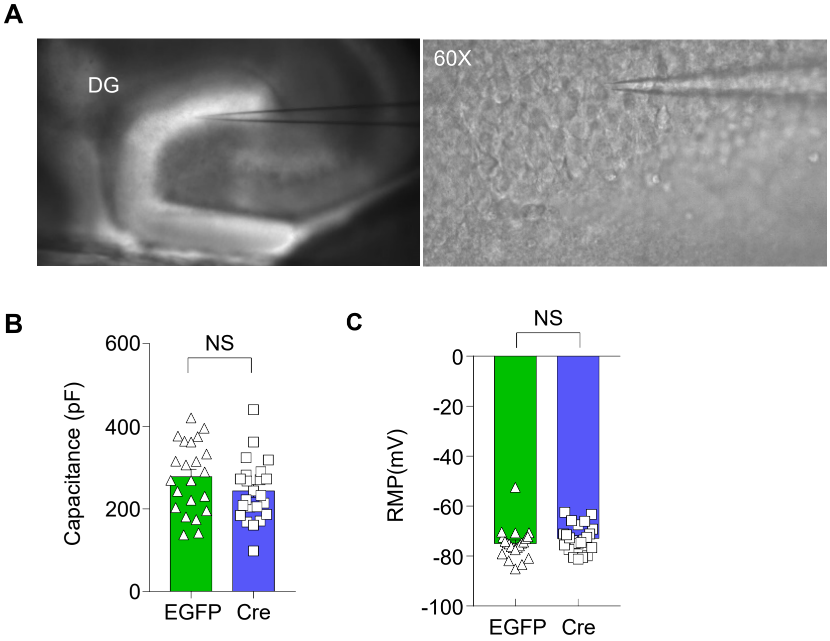

Fig. S5. Electrophysiological analyses of intrinsic properties of PLCβ1-depleted neurons from the dentate gyrus (A) Fluorescent image of brain slice showing DG neurons expressing EGFP (left) and the magnified differential interference contrast (DIC) image with glass patch-pipette (right). (B-C) Bar graphs of measured membrane capacitance (B) and resting membrane potential (C; RMP). Each granule cell in the dentate gyrus was labeled with EGFP through viral transduction of the Cre-DIO system or a vector encoding EGFP (control) into PLCβ1-floxed mice (EGFP n=22 Cre n=23; NS, not significant, unpaired t-test). All data represent means ± s.e.m.

Fig. S6

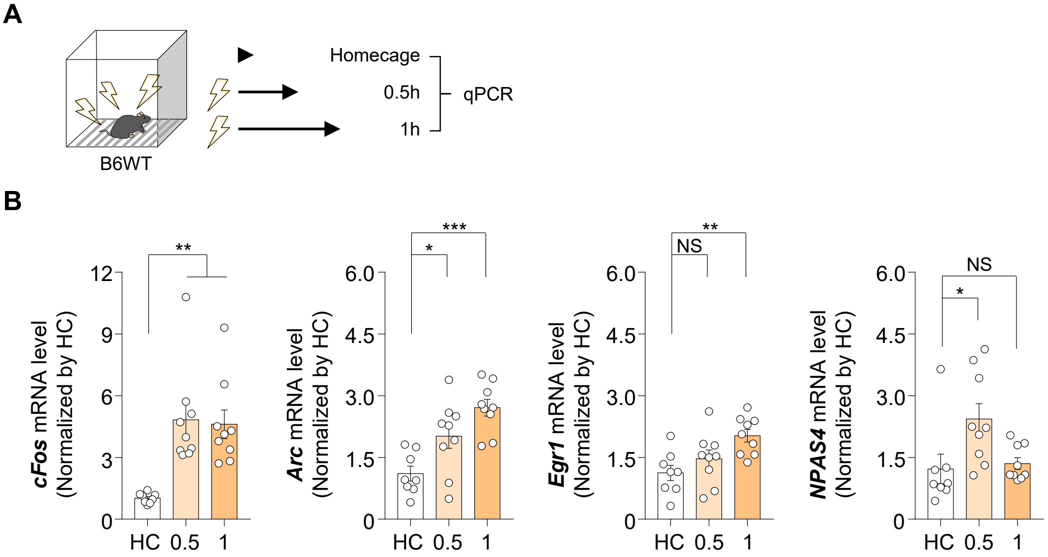

Fig. S6. Neural activity markers were assessed to measure neural activity
(A)**,** Schematic illustration of strategy for checking IEG induction over time. **b,** Graphs showing normalized mRNA levels (Home cage(HC) n=8, 0.5h n=10, 1h n=9; *p<0.05, **p<0.005, **p<0.001, one-way ANOVA with multiple comparisons). All data represent means ± s.e.m.

Fig. S7

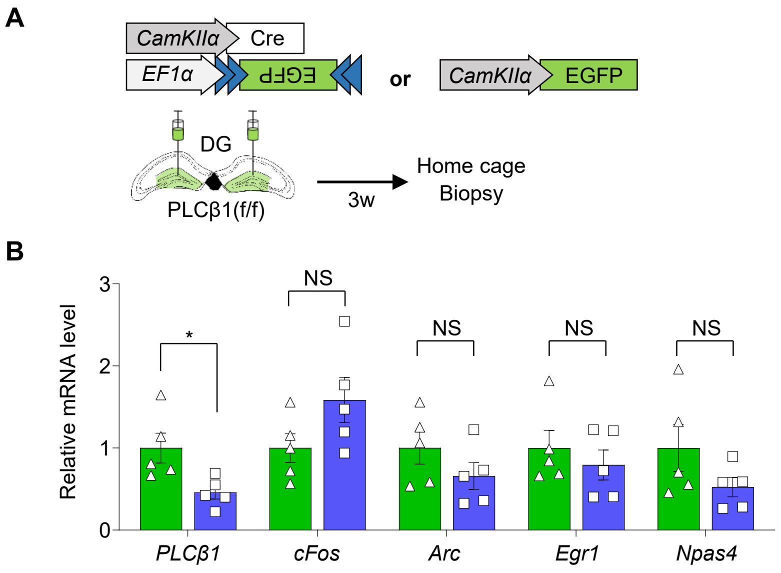

Fig. S7. Assessment of neural activity under PLCβ1 depletion in the home cage

(A, B) Schematic illustration (A) and bar graphs (B) showing the mRNA levels of IEGs under PLCβ1 depletion without contextual fear input (EGFP n=5, Cre n=5; *$p<0.05$, NS, not significant; unpaired t-test). All data represent means ± s.e.m.

Fig. S8

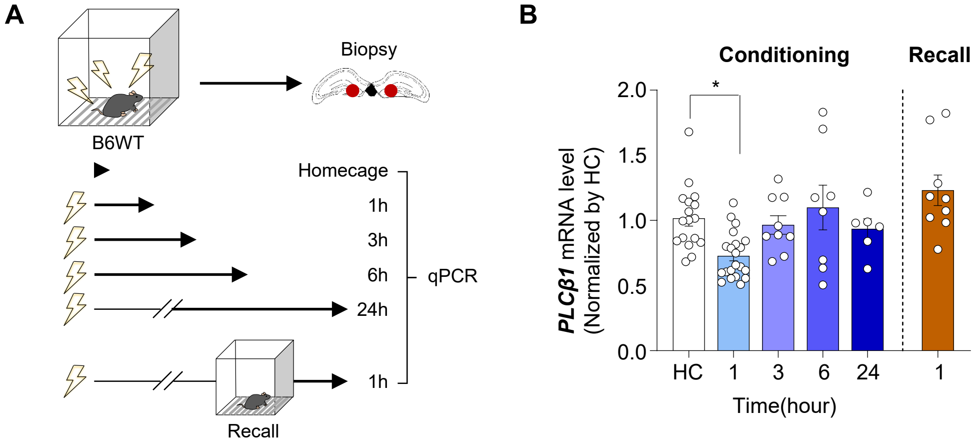

Fig. S8. Changes in PLCβ1 mRNA level of the dentate gyrus following contextual fear conditioning and recall (A) Schematic depicting the strategy for quantifying PLCβ1 mRNA levels at various times following fear learning. (B) Graph showing fluctuations in the relative PLCβ1 transcript level. (Homecage (HC) n=9, 1h n=13, 3h n=9, 6h n=8, 24h n=6, Recall 1h n=9; *$p<0.05$, one-way ANOVA). All data represent means ± s.e.m.

Fig. S9

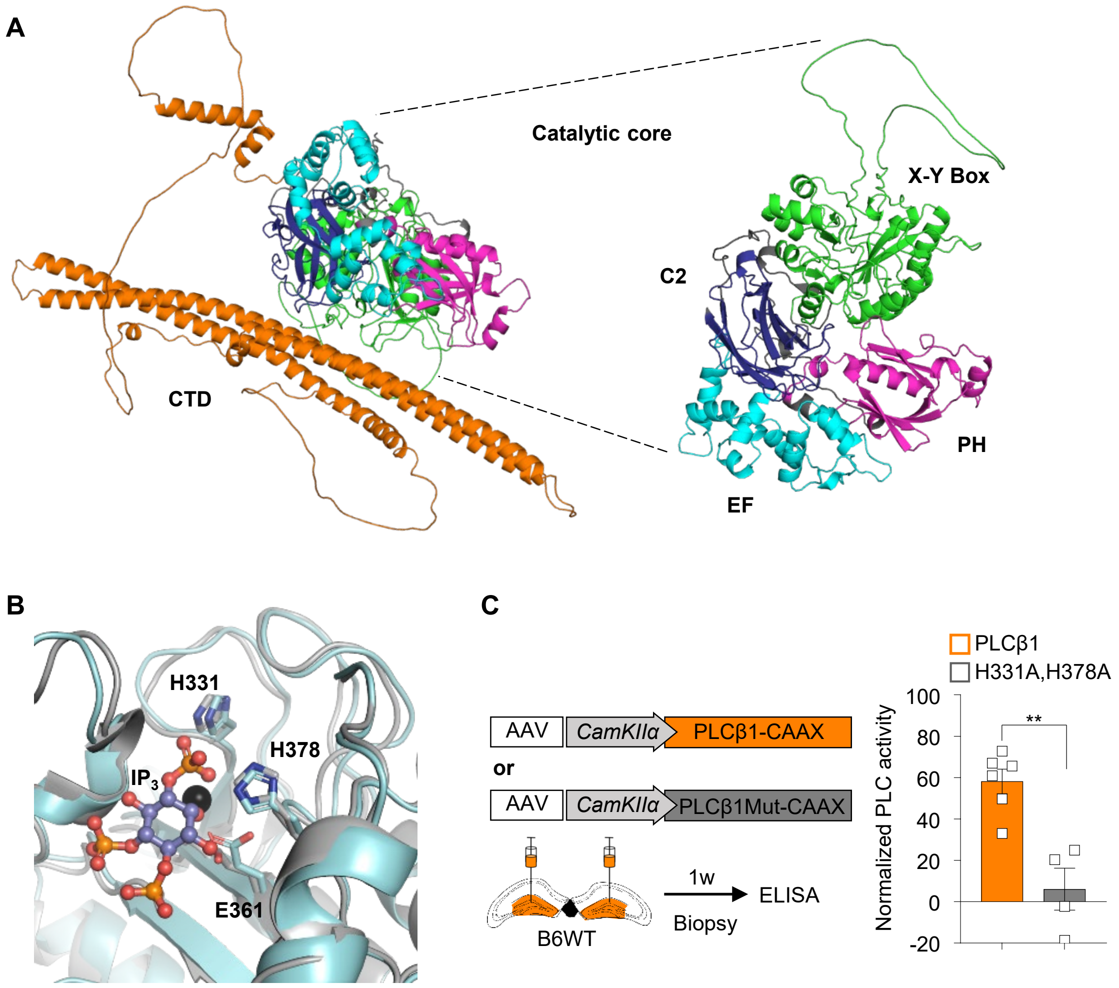

Fig. S9. Construct design for generation of constitutively active and inactive PLCβ1

(A) The 3D structure of full-length PLCβ1 was predicted by AlphaFold2 using ColabFold v1.5.2. (B) Aligned structural diagram of PLCβ1 (sky) and PLCβ3 (gray), indicating catalytic residues of PLCβ1. The structure of PLCβ1 was predicted by AlphaFold2, while those of PLCβ3 and IP3 were derived from PDB ID 4GNK and 1DJX, respectively. The enzymatically active residues, H331, H378A, and E361, are displayed as sticks and the active site Ca2+ is shown as a black sphere. (C) Verification of the PLC enzymatic activity of PLCβ1CAAX and its point-mutated form in mouse brain, as assessed by measuring the IP1 level 2 hours after intraperitoneal injection with LiCl (50mM). (PLCβ1(1-816)-CAAX n=6, PLCβ1(1-816)(H331A,H378A)-CAAX n=4;**$p<0.005$, unpaired t-test). All data represent means ± s.e.m.

Fig. S10

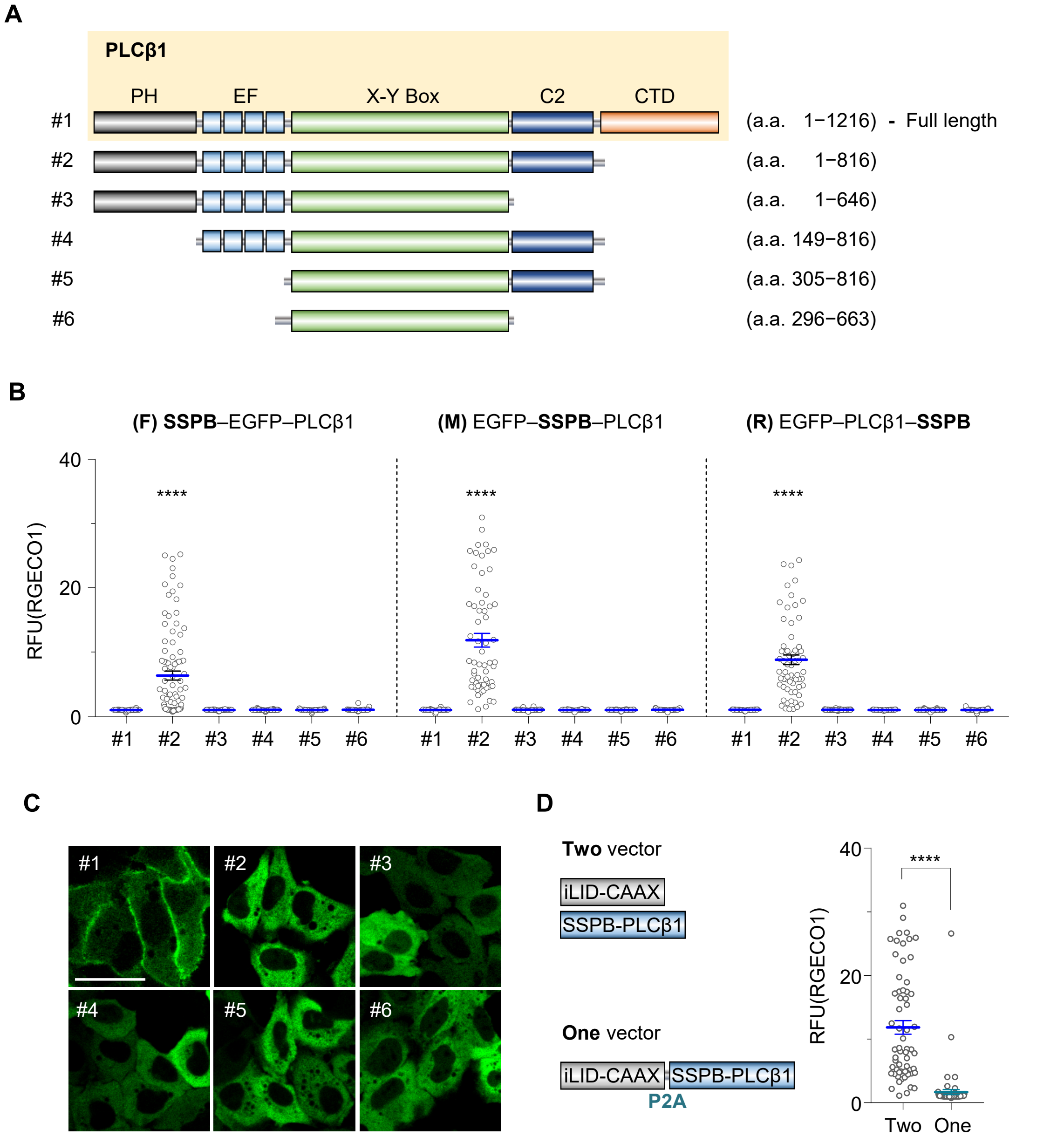

Fig. S10. Constructs for generating optogenetic system for PLCβ1 activation and Ca2+ response under blue-light illumination

(A) Schematic illustration of each truncated PLCβ1 construct. (B) Graph showing maximal fold change of R-GECO1 under blue-light stimulation of SSPB-fused PLCβ1 variants with iRFP682-iLID-CAAX(KRas4B tail) in three different groups: F (#1 n=60, #2 n=83, #3 n=72, #4 n=95, #5 n=59, #6 n=53), M (#1 n=68, #2 n=62, #3 n=68, #4 n=78, #5 n=68, #6 n=57), and R (#1 n=59, #2 n=64, #3 n=107, #4 n=54, #5 n=75, #6 n=53). (**** P<0.0001, one-way ANOVA). (C) Confocal images showing subcellular localization of each variant tagged with EGFP at the N-terminus. Scale bar=50 µm. (D) A relatively robust increase of R-GECO1 intensity in two-vector system compared to one-vector system, achieved through polycistronic expression by insertion of P2A peptide (Two-vector n=62, One-vector n=69, unpaired t-test). Scale bar=100 µm. All data represent means ± s.e.m.

Fig. S11

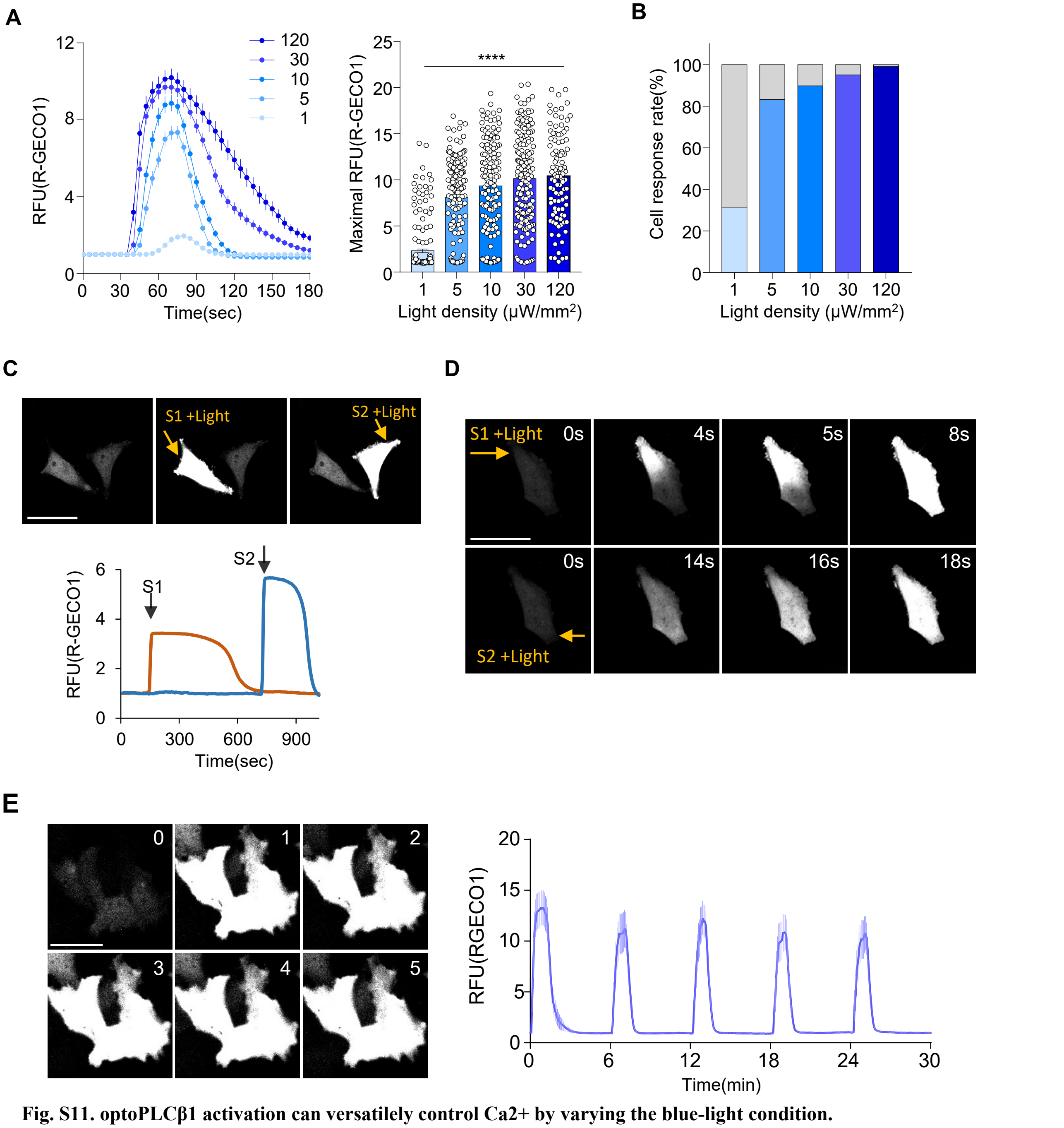

Fig. S11. optoPLCβ1 activation can versatilely control Ca2+ by varying the blue-light condition.
(A-B) Efficiency of Ca2+ increase under different intensity levels of blue light (a; ****p<0.0001, one-way ANOVA) and percentages of cells responding under each blue-light condition (B). The screened levels were (at 488 nm): 120 μW/mm² n=193, 30 μW/mm² n=155, 10 μW/mm² n=148, 5 μW/mm² n=180, 1 μW/mm² n=100). (C-D) Confocal images and graph showing Ca2+ increases induced by intercellular (C) and subcellular (D) activation of PLCβ1 signaling. For local stimulation, a single 0.15-μW laser pulse was delivered into a 5-μm-diameter circular region. Stimulated regions are indicated by yellow arrows. (E) Representative images and graph showing repetitive (five times) activation of PLCβ1 signaling (time-lapse imaging taken at 5-second intervals during 1 minute for activation and 5 minutes for the decline to baseline; 120 μW/mm2, n=25). All experiments were conducted in HeLaEG after lipofection of optoPLCβ1 and R-GECO1. Scale bar=50 μm. All data represent means ± s.e.m.

Fig. S12

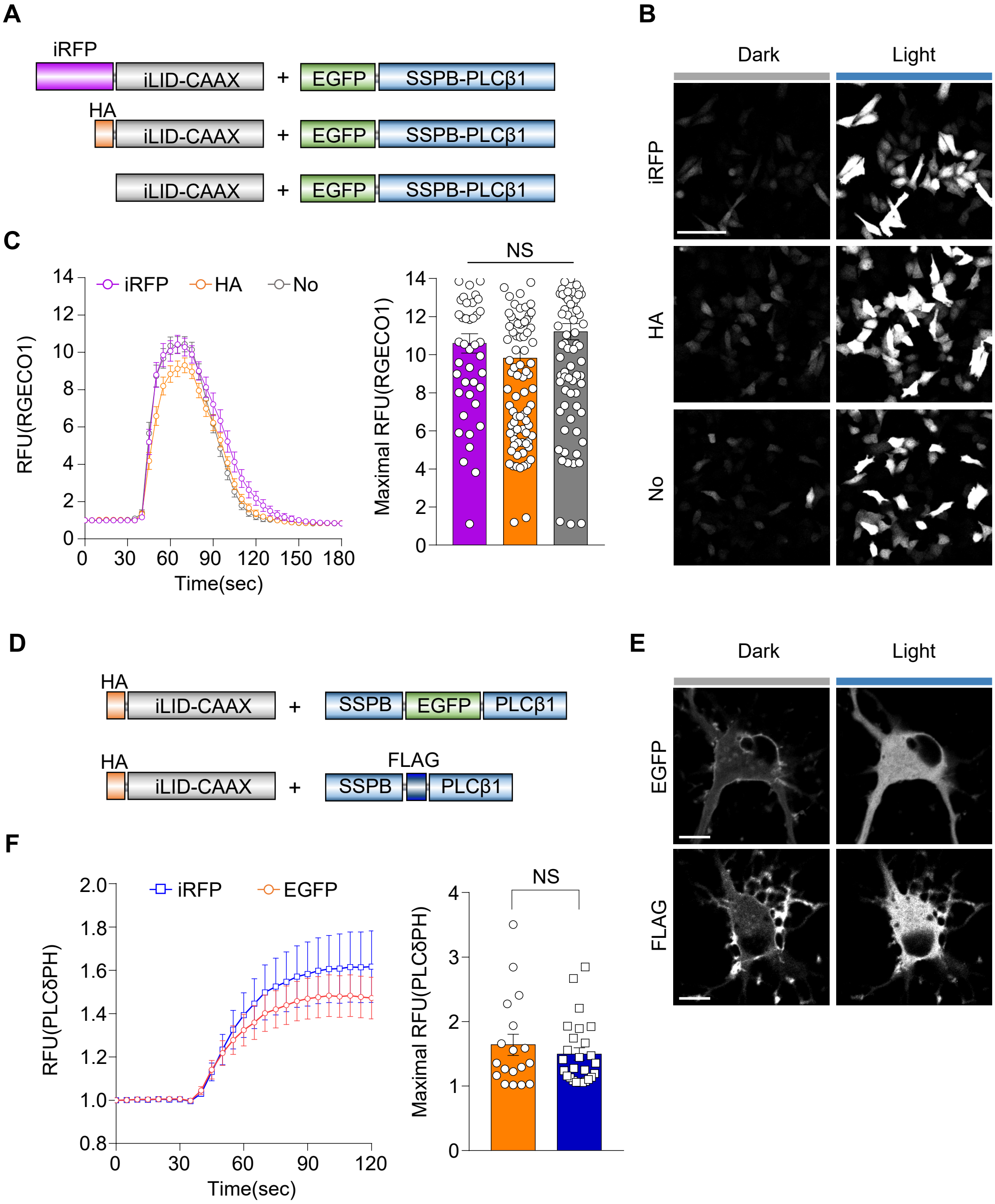

Fig. S12. Confirmation that optogenetic activation of PLCβ1 is not changed by replacement of fluorescent proteins with tag peptides to facilitate viral packaging.
(A) Schematic illustration showing each pairing of iLID-CAAX used to substitute iRFP682 with the HA tag. (B) Representative confocal images showing the Ca2+ increase observed in HeLa cells co-expressing each optoPLCβ1 and R-GECO1. Scale bar=100 μm. (C) Time-lapse graph and maximal fold change of R-GECO1 (iRFP n=92, HA n=98, no tag n=47; one-way ANOVA). (D) Schematic illustration showing each pairing of SSPB-PLCβ1 used to substitute the EGFP with the FLAG tag. (E) Representative confocal images showing translocation of (PLCδ)PH in hippocampal cultured neurons expressing each optoPLCβ1. Scale bar=10 μm. (F) Time-lapse graph and maximal fold change of mCherry-(PLCδ)PH (EGFP n=18, FLAG n=26; unpaired t-test). All data represent means ± s.e.m.

Fig. S13

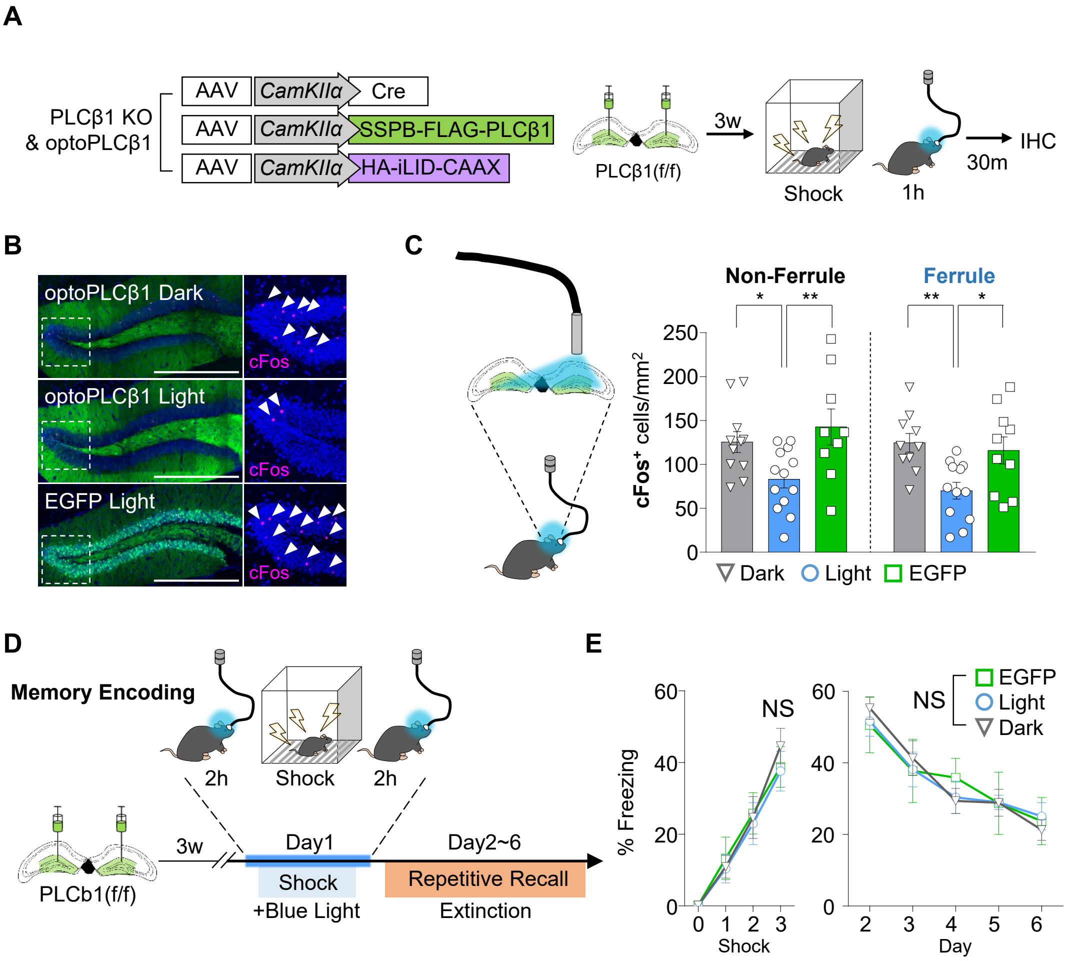

Fig. S13. Suppression of neural activity by optogenetic PLCβ1 activation in mice subjected to PLCβ1 knockout in the dentate gyrus

(A) Schematic of strategy for injecting Cre-recombinase for conditional deletion of PLCβ1 and optoPLCb1 for signaling activation. Mice received foot shocks, PLCβ1 was activated for 1 hour, and the mice were rested in their home cages for 30 min and then sacrificed. (B) Representative images showing PLCβ1 expression and cFos labeling (white arrows). Scale bar=500 µm. (C) Unilateral stimulation of optoPLCβ1 was sufficient to bilaterally activate optogenetic PLCβ1 (Non-ferrule Dark n=10, Light n=12, EGFP n=10; Ferrule Dark n=11, Light n=13, EGFP n=9; $^*p<0.05$, $^{**}p<0.005$ one-way ANOVA). (D) Schematic of strategy for optogenetic PLCβ1 activation during memory encoding. (E) Graphs showing that there was no significant change in fear learning or fear memory retrieval. (Dark n=9, Light n=8, EGFP n=4; two-way repeated measures ANOVA). All data represent means ± s.e.m.

Table. S1. Primer list for qPCR

Gene	Forward	Reverse	Ref.
cFos	GGGAGGACCTTACCTGTTCG	AGGCCAGATGTGGATGCT T	28
Arc	TACCGTTAGCCCCTATGCCATC	TGATATTGCTGAGCCTCAACTG	28
Egr1	CCTATGAGCACCTGACCACA	TCGTTTGGCTGGGATAACTC	28
Npas4	GCTATA CTCAGAAGGTCCAGAAGGC	TCAGAGAATGAGGGTAGCA CAGC	50
PLCβ1	GCCCCTGGAGATTCTGGAGT	GGGAGACTTGAGGTTCACCTTT	49
Gapdh	CTGAGTATGTCGTGGAGTCTACTGG	GTCATATTTCTCGTGGTTCACACC	

Table S2. Summary of statistical analyses for main figures

Figure	Group	Test	Statistics	P value	Pair-wise comparison
Fig. 1C	Day1 (EGFP vs Cre)	Two-way ANOVA<br>(Injection X shock)	<Injection> F(1, 15)= 0.3859<br><Shock> F(3, 45)=33.77<br><Interaction> F(3, 45)=1.38<br><Subject(matching)> F(15, 45)=3.054	p=0.5438<br>p<0.0001<br>p=0.2610<br>p=0.0019	Sidak's multiple comparisons<br>S0 EGFP vs Cre, t(60)=0.2637, p=0.9982<br>S1 EGFP vs Cre, t(60)=1.3640, p=0.5430<br>S2 EGFP vs Cre, t(60)=0.9567, p=0.8132<br>S3 EGFP vs Cre, t(60)=0.8191, p=0.8837
	Day2 (EGFP vs Cre)	Unpaired *t*-test	t(15)=0.7321	p=0.4754	N/A
	Day3 (EGFP vs Cre)	Unpaired *t*-test	t(15)=0.0293	p=0.9770	N/A
Fig. 1D	Day1 (EGFP vs Cre)	Two-way ANOVA<br>(Injection X shock)	<Injection> F(1, 11)= 0.4315<br><Shock> F(3, 33)=38.24<br><Interaction> F(3, 33)=0.5472<br><Subject(matching)> F(11, 33)=2.666	p=0.5248<br>p<0.0001<br>p=0.6535<br>p=0.0145	Sidak's multiple comparisons<br>S0 EGFP vs Cre, t(44)=0.0727, p>0.9999<br>S1 EGFP vs Cre, t(44)=0.1184, p>0.9999<br>S2 EGFP vs Cre, t(44)=0.2338, p=0.9989<br>S3 EGFP vs Cre, t(44)=1.3370, p=0.5376
	Day2 (EGFP vs Cre)	Unpaired *t*-test	t(6)=0.0727	p=0.9444	N/A
	Day3 (EGFP vs Cre)	Unpaired *t*-test	t(11)=0.2113	p=0.8365	N/A
Fig. 1E	Day1 (EGFP vs Cre)	Two-way ANOVA<br>(Injection X shock)	<Injection> F(1, 16)= 1.631<br><Shock> F(3, 48)=41.64<br><Interaction> F(3, 48)=1.238<br><Subject(matching)> F(16, 48)=3.7	p=0.2198<br>p<0.0001<br>p=0.3061<br>p=0.0002	Sidak's multiple comparisons<br>S0 EGFP vs Cre, t(64)=0.0029, p>0.9999<br>S1 EGFP vs Cre, t(64)=0.8904, p=0.8490<br>S2 EGFP vs Cre, t(64)=2.0790, p=0.1565<br>S3 EGFP vs Cre, t(64)=0.8468, p=0.8706
	Day2 (EGFP vs Cre)	Unpaired *t*-test	t(16)=3.008	p=0.0083	N/A
	Day3 (EGFP vs Cre)	Unpaired *t*-test	t(16)=2.424	p=0.0276	N/A
Fig. 1G	Day1 (EGFP vs Cre)	Two-way ANOVA<br>(Injection X shock)	<Injection> F(1, 22)= 0.5027<br><Shock> F(3, 66)=132.4<br><Interaction> F(3, 66)=1.409<br><Subject(matching)> F(22, 66)=3.639	p=0.4858<br>p<0.0001<br>p=0.2479<br>p<0.0001	Sidak's multiple comparisons<br>S0 EGFP vs Cre, t(88)=0.0039, p>0.9999<br>S1 EGFP vs Cre, t(88)=1.2440, p=0.6236<br>S2 EGFP vs Cre, t(88)=1.3580, p=0.5432<br>S3 EGFP vs Cre, t(88)=0.5066, p=0.9777
	Day2~6 (EGFP vs Cre)	Two-way ANOVA<br>(Injection X Day)	<Injection> F(1, 22)= 8.028<br><Day> F(4, 88)=34.00<br><Interaction> F(4, 88)=1.214<br><Subject(matching)> F(22, 88)=12.74	p=0.0097<br>p<0.0001<br>p=0.3044<br>p<0.0001	Sidak's multiple comparisons<br>Day2 EGFP vs Cre, t(110)=2.303, p=0.1107<br>Day3 EGFP vs Cre, t(110)=2.624, p=0.0487<br>Day4 EGFP vs Cre, t(110)=1.701, p=0.3820<br>Day5 EGFP vs Cre, t(110)=2.368, p=0.0943<br>Day6 EGFP vs Cre, t(110)=3.363, p=0.0053
Fig. 1H	Day2~6 (EGFP vs Cre)	Two-way ANOVA<br>(Injection X Day)	<Injection> F(1, 24)= 6.286<br><Day> F(4, 96)=41.53<br><Interaction> F(4, 96)=3.395<br><Subject(matching)> F(24, 96)=4.436	p=0.0193<br>p<0.0001<br>p=0.0121<br>p<0.0001	Sidak's multiple comparisons<br>Day2 EGFP vs Cre, t(120)=0<br>Day3 EGFP vs Cre, t(120)=2.060, p=0.1912<br>Day4 EGFP vs Cre, t(120)=1.134, p=0.7767<br>Day5 EGFP vs Cre, t(120)=2.037, p=0.2009<br>Day6 EGFP vs Cre, t(120)=3.860, p=0.0009
Fig. 2B	Input Resistance<br>(EGFP vs Cre)	Unpaired *t*-test	t(43)=2.716	p=0.0095	N/A
Fig. 2C	Rheobase<br>(EGFP vs Cre)	Unpaired *t*-test	t(30)=2.219	p=0.0342	N/A
Fig. 2E	IEG mRNA<br>(EGFP vs Cre)	Unpaired *t*-test<br>Unpaired *t*-test<br>Unpaired *t*-test<br>Unpaired *t*-test<br>Unpaired *t*-test	*PLCβ1* t(12)=7.078<br>*cFos* t(12)=5.855<br>*Arc* t(12)=0.7503<br>*Egr1* t(12)=2.244<br>*Npas4* t(12)=2.315	p<0.0001<br>p<0.0001<br>p=0.4675<br>p=0.0445<br>P=0.0391	N/A
Fig. 2G	IEG IHC<br>(EGFP vs Cre)	Unpaired *t*-test<br>Unpaired *t*-test<br>Unpaired *t*-test	cFos t(14)=4.496<br>Arc t(9)=0.6854<br>Egr1 t(14)=4.36	p=0.0005<br>p=0.5103<br>p=0.0007	N/A
Fig. 2J	RAM (EGFP vs Cre)	Unpaired *t*-test<br>Unpaired *t*-test	F-RAM t(10)=3.149<br>N-RAM t(9)=4.009	p=0.0103<br>p=0.0031	N/A
Fig. 3A	Day1 Shock<br>(EGFP vs Cre)	Two-way ANOVA<br>(Injection X shock)	<Injection> F(1, 11)= 0.3873<br><Shock> F(3, 33)=24.76<br><Interaction> F(3, 33)=0.08787<br><Subject(matching)> F(11, 33)=2.596	p=0.5464<br>p<0.0001<br>p=0.9662<br>p=0.0168	Sidak's multiple comparisons<br>S0 PLCβ1 vs inactive, t(44)=0.4118, p=0.9898<br>S1 PLCβ1 vs inactive, t(44)=0.6690, p=0.9409<br>S2 PLCβ1 vs inactive, t(44)=0.5320, p=0.9737<br>S3 PLCβ1 vs inactive, t(44)=0.0826, p>0.9999
	Day1 1hr STM<br>(EGFP vs Cre)	Unpaired *t*-test	t(11)=2.510	p=0.0290	N/A
Fig. 3C	Day2 Context A<br>(ArchT vs Cre only)	Unpaired *t*-test	t(13)=2.940	p=0.0115	N/A
	Day3 Context B<br>(ArchT vs Cre only)	Unpaired *t*-test	t(9)=2.778	p=0.0157	N/A
Fig. 3E	Day1 PLCβ1CAAX<br>(PLCβ1 vs inactive)	Two-way ANOVA<br>(Injection X shock)	<Injection> F(1, 13)= 1.755<br><Shock> F(3, 39)=64.77<br><Interaction> F(3, 39)=0.9807<br><Subject(matching)> F(13, 39)=2.140	p=0.2080<br>p<0.0001<br>p=0.9807<br>p=0.0336	Sidak's multiple comparisons<br>S0 PLCβ1 vs inactive, t(52)=0.0427, p>0.9999<br>S1 PLCβ1 vs inactive, t(52)=0.7055, p=0.9289<br>S2 PLCβ1 vs inactive, t(52)=0.7023, p=0.9300<br>S3 PLCβ1 vs inactive, t(52)=2.0540, p=0.1682

Figure	Group	Test	Statistics	P value	Pair-wise comparison
Fig. 3E	Day2~6<br>(PLCβ1 vs inactive)	Two-way ANOVA<br>(Injection X Day)	<Injection> F(1, 13)= 66.98<br><Day> F(4, 52)=13.35<br><Interaction> F(4, 52)=3.947<br><Subject(matching)> F(13, 52)=1.996	p<0.0001<br>p<0.0001<br>p=0.0072<br>p<0.0399	Sidak's multiple comparisons<br>Day2 PLCβ1 vs inactive, t(65)=6.539, p<0.0001<br>Day3 PLCβ1 vs inactive, t(65)=6.103, p<0.0001<br>Day4 PLCβ1 vs inactive, t(65)=5.099, p<0.0001<br>Day5 PLCβ1 vs inactive, t(65)=3.778, p=0.0017<br>Day6 PLCβ1 vs inactive, t(65)=2.091, p=0.1866
Fig. 3F	Day3 Context B<br>(PLCβ1 vs inactive)	Unpaired *t*-test	t(9)=2.492	p=0.0343	N/A
Fig. 4E	Basal activity	One-way ANOVA	F(2, 11)=37.49	P<0.0001	Tukey's multiple comparisons<br>Dark vs Cch, q(11)=9.744, p<0.0001<br>Dark vs EGFP, q(11)=0.7529, p=0.8573<br>Cch vs EGFP, q11)=11.13, p<0.0001
Fig. 4F	optoPLCβ1 activation	One-way ANOVA	F(3, 20)=4.388	p=0.0158	Dunnett's multiple comparisons<br>Dark vs 10min, q(20)=1.73, p=0.2294<br>Dark vs 30min, q(20)=2.92, p=0.0224<br>Dark vs 60min, q(20)=3.431, p=0.0072
Fig. 4I	activity (Dark vs Light)	Unpaired *t*-test	t(9)=2.681	p=0.0252	N/A
Fig. 5B	cFos (Dark vs Light)	Unpaired *t*-test	t(33)=2.739	p=0.0098	N/A
Fig. 5D	Day1 (Dark vs Light)	Two-way ANOVA<br>(Injection X shock)	<Injection> F(1, 11)= 0.1.086<br><Shock> F(3, 33)=40.3<br><Interaction> F(3, 33)=0.5007<br><Subject(matching)> F(11, 33)=3.132	p=0.3197<br>p<0.0001<br>p=0.6844<br>p=0.0054	Sidak's multiple comparisons<br>S0 Dark vs Light, t(44)=0.2899, p=0.9974<br>S1 Dark vs Light, t(44)=1.5690, p=0.4104<br>S2 Dark vs Light, t(44)=0.4492, p=0.9859<br>S3 Dark vs Light, t(44)=0.6706, p=0.9404
	Day2~6 (Dark vs Light)	Two-way ANOVA<br>(Injection X Day)	<Injection> F(1, 11)= 10.48<br><Day> F(4, 44)=14.48<br><Interaction> F(4, 44)=0.5989<br><Subject(matching)> F(11, 44)=2.944	p=0.0079<br>p<0.0001<br>p=0.6654<br>p=0.0053	Sidak's multiple comparisons<br>Day2 Dark vs Light, t(55)=2.003, p=0.2266<br>Day3 Dark vs Light, t(55)=2.836, p=0.0315<br>Day4 Dark vs Light, t(55)=2.1, p=0.1859<br>Day5 Dark vs Light, t(55)=2.505, p=0.0739<br>Day6 Dark vs Light, t(55)=1.095, p=0.8045
Fig. 5F	Day1 (Dark vs Light)	Two-way ANOVA<br>(Injection X shock)	<Injection> F(1, 14)= 0.5340<br><Shock> F(3, 42)=65.86<br><Interaction> F(3, 42)=2.379<br><Subject(matching)> F(14, 42)=3.861	p=0.4770<br>p<0.0001<br>p=0.0833<br>p=0.0003	Sidak's multiple comparisons<br>S0 Dark vs Light, t(56)=0.0023, p>0.9999<br>S1 Dark vs Light, t(56)=0.5674, p=0.9667<br>S2 Dark vs Light, t(56)=0.5984, p=0.9597<br>S3 Dark vs Light, t(56)=2.1640, p=0.1319
	Day2~6 (Dark vs Light)	Two-way ANOVA<br>(Injection X Day)	<Injection> F(1, 14)= 0.2904<br><Day> F(4, 56)=27.11<br><Interaction> F(4, 56)=0.8476<br><Subject(matching)> F(14, 56)=5.559	p=0.5984<br>p<0.0001<br>p=0.5011<br>p<0.0001	Sidak's multiple comparisons<br>Day2 Dark vs Light, t(70)=0.1362, p>0.9999<br>Day3 Dark vs Light, t(70)=0.9783, p=0.8663<br>Day4 Dark vs Light, t(70)=1.0920, p=0.8048<br>Day5 Dark vs Light, t(70)=0.5315, p=0.9893<br>Day6 Dark vs Light, t(70)=0.4102, p=0.9968
Fig. 5H	Day3 (Dark vs Light)<br>Day4 (Dark vs Light)	Unpaired *t*-test<br>Unpaired *t*-test	t(10)=3.508<br>t(10)=0.7955	p=0.0056<br>p=0.4448	N/A<br>N/A
Fig. 5I	Day3 (Dark vs Light)<br>Day4 (Dark vs Light)	Unpaired *t*-test<br>Unpaired *t*-test	t(10)=0.05915<br>t(10)=0.1869	p=0.9540<br>p=0.8555	N/A<br>N/A
Fig. 6B	PTSD (RES vs SUS)	Two-way ANOVA<br>(Phenotype X Day)	<Phenotype> F(1, 16)=3.533<br><Day> F(4, 64)=4.236<br><Interaction> F(4, 64)=13.39<br><Subject(matching)> F(16, 64)=3.533	p<0.0001<br>p=0.0042<br>p<0.0001<br>p=0.0002	Sidak's multiple comparisons<br>Day2 RES vs SUS, t(80)=0<br>Day3 RES vs SUS, t(80)=6.739, p<0.0001<br>Day4 RES vs SUS, t(80)=6.8, p<0.0001<br>Day5 RES vs SUS, t(80)=6.549, p<0.0001<br>Day6 RES vs SUS, t(80)=6.554, p<0.0001
Fig. 6C	PTSD (RES vs SUS)	Unpaired *t*-test	t(16)=2.352	p=0.0318	N/A
Fig. 6E	Day3<br>(PLCβ1 vs inactive)	Unpaired *t*-test	t(15)=0.2.272	p=0.0382	N/A
Fig. 6F	Day3~7<br>(PLCβ1 vs inactive)	Two-way ANOVA<br>(Injection X Day)	<Injection> F(1, 15)=4.972<br><Day> F(4, 60)=34.06<br><Interaction> F(4, 60)=3.116<br><Subject(matching)> F(15,60)=3.876	p=0.0415<br>p<0.0001<br>p=0.0214<br>p<0.0001	Sidak's multiple comparisons<br>Day2 PLCβ1 vs inactive, t(75)=3.584, p=0.0030<br>Day3 PLCβ1 vs inactive, t(75)=2.359, p=0.1003<br>Day4 PLCβ1 vs inactive, t(75)=1.221, p=0.7219<br>Day5 PLCβ1 vs inactive, t(75)=0.2041, p=0.9999<br>Day6 PLCβ1 vs inactive, t(75)=0.4532, p=0.9949
Fig. 6H	Day3 (Dark vs Light)	Unpaired *t*-test	t(22)=2.241	p=0.0354	N/A
Fig. 6I	Day3~7 (Dark vs Light)	Two-way ANOVA<br>(Injection X Day)	<Injection> F(1, 23)= 4.460<br><Day> F(4, 92)=76.42<br><Interaction> F(4, 92)=0.1247<br><Subject(matching)> F(23, 92)=17.81	p=0.0458<br>p<0.0001<br>p=0.1247<br>p<0.0001	Sidak's multiple comparisons<br>Day2 Dark vs Light, t(115)=2.535, p=0.0615<br>Day3 Dark vs Light, t(115)=2.545, p=0.0598<br>Day4 Dark vs Light, t(115)=1.085, p=0.8068<br>Day5 Dark vs Light, t(115)=1.433, p=0.5680<br>Day6 Dark vs Light, t(115)=1.945, p=0.2433

Table S3. Summary of statistical analyses for Supplementary Data

Figure	Group	Test	Statistics	P value	Pair-wise comparison
Fig. S2B	Day1 (EGFP vs Cre)	Two-way ANOVA<br>(Injection X shock)	<Injection> F(1, 11)= 1.445<br><Shock> F(3, 33)=54.72<br><Interaction> F(3, 33)=0.8266<br><Subject(matching)> F(11, 39)=1.556	p=0.2530<br>p<0.0001<br>p=0.4887<br>p=0.1588	Sidak's multiple comparisons<br>S0 Cre vs EGFP, t(44)=0.4454, p>0.9999<br>S1 Cre vs EGFP, t(44)=0.7786, p>0.9999<br>S2 Cre vs EGFP, t(44)=1.6100, p=0.4581<br>S3 Cre vs EGFP, t(44)=0.8762, p>0.9999
	Day2 (EGFP vs Cre)	Unpaired *t*-test	t(11)=0.8520	p=0.4124	N/A
	Day3 (EGFP vs Cre)	Unpaired *t*-test	t(11)=0.01987	p=0.9854	N/A
Fig. S3B	Total distance<br>(Cre vs EGFP)	Unpaired *t*-test	t(16)=0.7338	p=0.4707	N/A
	Time in center<br>(Cre vs EGFP)	Unpaired *t*-test	t(16)=1.223	p=0.2392	N/A
	Latency<br>(Cre vs EGFP)	Unpaired *t*-test	t(16)=0.4385	p=0.6669	N/A
Fig. S4B	Day1 (EGFP vs Cre)	Two-way ANOVA<br>(Injection X shock)	<Injection> F(1, 13)= 0.2602<br><Shock> F(3, 39)=42.68<br><Interaction> F(3, 39)=0.9154<br><Subject(matching)> F(13, 39)=4.038	p=0.6185<br>p<0.0001<br>p=0.4424<br>p=0.0003	Sidak's multiple comparisons<br>S0 EGFP vs Cre, t(52)=0.0296, p>0.9999<br>S1 EGFP vs Cre, t(52)=1.3240, p=0.5721<br>S2 EGFP vs Cre, t(52)=0.5569, p=0.9689<br>S3 EGFP vs Cre, t(52)=0.3058, p=0.9967
Fig. S4C	Day2 (EGFP vs Cre)	Unpaired *t*-test	t(13)=2.272	p=0.0407	N/A
Fig. S4D	Day3 (EGFP vs Cre)	One-way ANOVA	F(3, 26)=24.48	p<0.0001	Sidak's multiple comparisons<br>(EGFP) -tone vs +tone, t(26)=5.788, p<0.0001<br>(Cre) -tone vs +tone, t(26)=6.321, p<0.0001<br>(-Tone) EGFP vs Cre, t(26)=0.0533, p>0.9999<br>(+Tone) EGFP vs Cre, t(26)=0.0755, p>0.9999
Fig. S5B	Capacitance<br>(EGFP vs Cre)	Unpaired *t*-test	t(43)=1.448	p=0.1549	N/A
Fig. S5C	RMP<br>(EGFP vs Cre)	Unpaired *t*-test	t(43)=1.142	p=0.2598	N/A
Fig. S6B	IEG(*cFos*)	One-way ANOVA	F(2, 23)=10.56	p=0.0001	Tuckey's multiple comparisons<br>Home vs 0.5hr, q(23)=5.840, p=0.0011<br>Home vs 1hr, q(23)=5.512, p=0.0020<br>0.5hr vs 1hr, q(23)=0.3383, p=0.9690
	IEG(*Arc*)	One-way ANOVA	F(2, 23)=11.36	p=0.0004	Tuckey's multiple comparisons<br>Home vs 0.5hr, q(23)=3.816, p=0.0331<br>Home vs 1hr, q(23)=6.736, p=0.0002<br>0.5hr vs 1hr, q(23)=3.009, p=0.1062
	IEG(*Egr1*)	One-way ANOVA	F(2, 23)=5.989	p=0.0081	Tuckey's multiple comparisons<br>Home vs 0.5hr, q(23)=1.834, p=0.4113<br>Home vs 1hr, q(23)=4.812, p=0.0065<br>0.5hr vs 1hr, q(23)=3.079, p=0.0966
	IEG(*NPAS4*)	One-way ANOVA	F(2, 23)=4.808	p=0.0001	Tuckey's multiple comparisons<br>Home vs 0.5hr, q(23)=3.929, p=0.0278<br>Home vs 1hr, q(23)=0.4267, p=0.9512<br>0.5hr vs 1hr, q(23)=3.610, p=0.0452
Fig. S7B	IEG mRNA in HC<br>(EGFP vs Cre)	Unpaired *t*-test<br>Unpaired *t*-test<br>Unpaired *t*-test<br>Unpaired *t*-test<br>Unpaired *t*-test	*PLCβ1* t(8)=2.750<br>*cFos* t(8)=1.790<br>*Arc* t(8)=1.342<br>*Egr1* t(8)=0.7343<br>*Npas4* t(8)=1.556	P=0.0250<br>P=0.1113<br>p=0.2165<br>p=0.4838<br>P=0.1583	N/A
Fig. S8B	PLCβ1 mRNA level<br>(Time)	One-way ANOVA	F(5, 64)=5.390	p=0.0003	Dunnett's multiple comparisons<br>HC vs 1h, q(64)=3.238, p=0.0.0090<br>HC vs 3h, q(64)=0.4563, p=0.9918<br>HC vs 6h, q(64)=0.7120, p=0.9438<br>HC vs 24h, q(64)=0.6324, p=0.9652<br>HC vs Recall 1h, q(64)=1.917, p=0.2326
Fig. S9C	IP1 production<br>(PLCβ1 vs Mutant)	Unpaired *t*-test	t(8)=4.762	p=0.0014	N/A
Fig. S10B	Truncated PLCβ1(F)<br>Truncated PLCβ1(M)<br>Truncated PLCβ1(R)	One-way ANOVA	F(5, 446)=43.55<br>F(5, 395)=111.5<br>F(5, 406)=115.9	p<0.0001<br>p<0.0001<br>p<0.0001	N/A
Fig. S10D	Vector(One vs Two)	Unpaired *t*-test	t(129)=9.24	p<0.0001	N/A
Fig. S11B	Light sensitivity	One-way ANOVA	F(4, 770)=100.2	p<0.0001	N/A
Fig. S12C	iLID-CAAX tag<br>(None vs iRFP vs HA)	One-way ANOVA	F(2, 234)=100.2	p=0.0556	N/A
Fig. S12F	SSPB-PLCβ1 tag<br>(EGFP vs FLAG)	Unpaired *t*-test	t(42)=0.7974	p=0.4297	N/A

Figure	Group	Test	Statistics	P value	Pair-wise comparison
Fig. S13C	optoPLCβ1 activity(Non-FR)<br>(EGFP vs Dark vs Light)	One-way ANOVA	F(2, 30)=5.28	p=0.0108	Tukey’s multiple comparisons<br>Dark vs Light, q(30)=3.26, p=0.0704<br>Dark vs EGFP, q(30)=1.207, p=0.6733<br>Light vs EGFP, q(30)=4.331, p=0.0124
	optoPLCβ1 activity (FR)<br>(EGFP vs Dark vs Light)	One-way ANOVA	F(2, 29)=6.41	p=0.0049	Tukey’s multiple comparisons<br>Dark vs Light, q(29)=4.648, p=0.0073<br>Dark vs EGFP, q(29)=0.7133, p=0.8698<br>Light vs EGFP, q(29)=3.903, p=0.0260
Fig. S13E	Day1<br>(Dark vs Light vs EGFP)	Two-way ANOVA<br>(Injection X shock)	<Injection> F(2, 20)=0.5188<br><Shock> F(3, 60)=71.37<br><Interaction> F(6, 60)=0.3806<br><Subject(matching)> F(20, 60)=3.791	p=0.6030<br>p<0.0001<br>p=0.8886<br>p<0.0001	N/A
	Day2~6<br>(Dark vs Light vs EGFP)	Two-way ANOVA<br>(Injection X Day)	<Injection> F(2, 20)=0.6059<br><Day> F(4, 80)=46.96<br><Interaction> F(8, 80)=0.4254<br><Subject(matching)> F(20, 80)=13.6	p=0.5553<br>p<0.0001<br>p=0.9026<br>p<0.0001	N/A
